# The conserved nematode pheromone ascr#18 primes plant immunity

**DOI:** 10.1038/s42003-026-10211-1

**Published:** 2026-05-06

**Authors:** Murli Manohar, Andrea Sistenich, Shoashuai Liu, Shine Baby, Shiyan Chen, Wim Dejonghe, Anshu Kumari, Emily Luna, Sophie Levecque, Patricia M. Manosalva, Jan E. Leach, Xiaohong Wang, Aardra Kachroo, Karl-Heinz Kogel, Uwe Conrath, Frank C. Schroeder, Daniel F. Klessig

**Affiliations:** 1 Boyce Thompson Institute, Ithaca, NY USA; 2 Department of Molecular Plant Physiology, RWTH Aachen University, Aachen, Germany; 3 Research Center for BioSystems, Land Use, and Nutrition, Justus Liebig University, Giessen, Germany; 4 Department of Plant Pathology, University of Kentucky, Lexington, KY USA; 5 School of Integrative Plant Science, Cornell University, Ithaca, NY USA; 6 Ascribe Bioscience, Ithaca, NY USA; 7 Department of Agricultural Biology, Colorado State University, Fort Collins, CO USA; 8 Department of Plant Pathology and Microbiology, University of California, Riverside, CA USA; 9 Robert W. Holley Center for Agriculture and Health, Agricultural Research Service, US Department of Agriculture, Ithaca, NY USA; 10 Present Address: Institute of Plant Molecular Biology (CNRS), University of Strasbourg, Strasbourg, France

**Keywords:** Pattern recognition receptors in plants, Agriculture, Microbe

## Abstract

Plants sense a diverse array of small molecules and macromolecules derived from their natural environment, including diverse microbe-associated molecular patterns (MAMPs) that can trigger defense responses. Several MAMPs have been shown to prime plants for enhanced defense, providing extended protection against pathogens with minimal fitness costs. However, the extent to which conserved small molecule signatures of other phyla contribute to priming of plant defenses is unclear. Here, we demonstrate that exposure of seeds or plants to the ascaroside ascr#18, a pheromone secreted by plant-parasitic and free-living soil nematodes, primes immune genes for enhanced expression upon pathogen challenge, thereby increasing resistance to diverse microbial pathogens. We further show that ascr#18-induced priming is associated with the formation of open chromatin in the regulatory regions of defense genes. Defense priming and disease protection by ascr#18 is compromised in *Arabidopsis* mutants defective in the receptor of ascr#18, the leucine-rich repeat receptor-like kinase NILR1. Defense priming by ascr#18 is retained under field conditions, demonstrating potential of ascaroside treatment as a crop protection strategy to reduce pesticide usage. Our findings provide insight into the molecular basis of defense priming by ascr#18 and demonstrate how evolutionarily conserved small molecule signatures of plant-associated macrobiota modulate immunity.

## Abstract (web summary)

A conserved nematode pheromone, ascr#18, primes plant immunity via chromatin remodeling dependent on the receptor kinase NILR1, enabling enhanced defense gene activation and broad-spectrum disease resistance across multiple plant species.

## Introduction

**Fig. 1:**
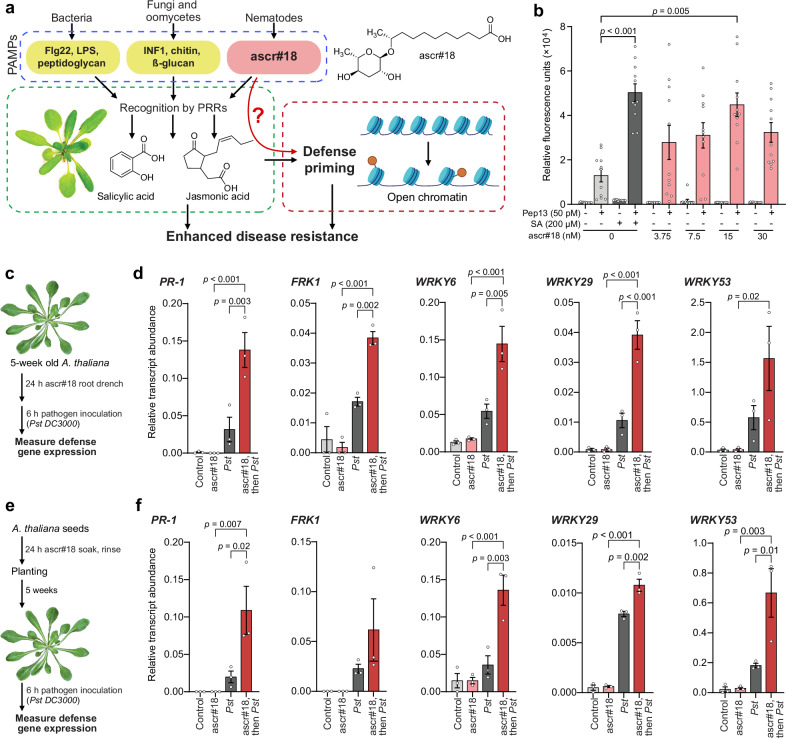
Ascr#18 primes defense responses in cultured parsley cells and *Arabidopsis* plants*.* **a** Model for general roles of MAMPs in triggering enhanced disease resistance in plants. **b** Pretreatment with ascr#18 primes suspension-cultured parsley cells for enhanced furanocoumarin secretion induced by Pep13. Three-day-old parsley cell cultures were treated with a control solution, the indicated concentrations of ascr#18, or 200 µM SA. After 24 h, 50 pM of Pep13 was added. Twenty-four hours later, fluorescence of secreted furanocoumarins was quantified. Data are mean ± SEM (*n* = 11). **c** Scheme for priming of 5-week-old *Arabidopsis* plants (Col-0) with ascr#18 (1 µM) via root drench. After 24 h, plants were inoculated with virulent *Pst* DC3000. **d** mRNA transcript levels of defense-response genes were measured by RT-qPCR in leaf tissue collected 6 h after inoculation. Transcript levels were normalized to *ACTIN2*. Data are mean ± SEM (*n* = 3). **e** Scheme for priming via soaking of *Arabidopsis* (Col-0) seeds with ascr#18 (1 µM). After 24 h, the seeds were rinsed with water and planted. Five weeks later, plants were inoculated with *Pst* DC3000. **f** mRNA transcript levels of defense-response genes were measured as in (**d**), and normalized to *ACTIN2*. Data are mean ± SEM (*n* = 3). In **b**, **d**, **f**, adjusted *p*-values were calculated using one-way ANOVA and Tukey multiple comparisons post hoc tests. Source data are provided as a Source data file.

Evidence is accumulating to suggest that the pervasive influence of microbiota on immune responses and disease susceptibility in both plants and animals is partly due to epigenetic reprogramming that recalibrate host transcriptional responses^1–3^. Immune responses in both plants and animals can be activated through pattern recognition receptors (PRRs) that have evolved to detect unique molecular signatures of pathogens^4,5^ (Fig. 1a). In plants, the perception of these microbe-associated molecular patterns (MAMPs) triggers the production of reactive oxygen species, the activation of specific mitogen-activated protein kinases (MAPKs), and the induction of defense pathways mediated by, for example, salicylic acid (SA), *N*-hydroxypipecolic acid, jasmonic acid, and ethylene^6–12^.

The induction of defense responses in plants is commonly linked to reduced growth, often referred to as the “growth versus defense/immunity tradeoff”. This phenomenon is thought to arise from the reallocation of a plant’s limited energy and nutrient resources toward defense at the expense of growth^13,14^. However, a subset of MAMPs and chemical agents naturally produced by plants, e.g., β-aminobutyric acid^15^ and *N*-hydroxypipecolic acid^16^, has been shown to prime plant immune systems, enhancing their defense capabilities against subsequent challenges^1,2,17^. As opposed to direct activation of defense responses, defense priming does not typically incur a significant fitness cost^1,18,19^. Primed plants exhibit faster and/or stronger responses to pathogen attacks, resulting in enhanced resistance compared to non-primed plants^1,2,20–23^.

Defense priming in plants and animals can be the result of covalent chromatin modifications, including alterations in DNA methylation and histone modifications (particularly histone acetylation, which can weaken histone-DNA interactions), leading to increased chromatin accessibility in promoter regions of immune genes^20,23–30^ (Fig. 1a). Additionally, in some priming systems, enhanced immune responses have been associated with increased PRR abundance^31^ and dormant cellular signaling enzymes (e.g., MAPKs) that can be rapidly activated upon subsequent pathogen attack^4^.

Recent work demonstrated that ascarosides, a family of highly conserved nematode pheromones^32–34^, function similar to MAMPs. At low micromolar and nanomolar concentrations, ascaroside pheromones were shown to elicit immune responses across a wide range of plant species, including both monocots and dicots^35–38^. The ascaroside most abundantly produced by plant-parasitic nematodes, ascr#18, is perceived by a specific PRR, a leucine-rich repeat receptor-like kinase (LRR-RLK) named nematode-induced LRR-RLK1 (NILR1)^39^. In addition, it was shown that a structurally distinct ascaroside produced by animal-parasitic nematodes influences mammalian immune responses^40^, indicating that these molecules are perceived as a conserved molecular signature of nematodes in both animals and plants, akin to many MAMPs. In plants, exposure to low concentrations of ascr#18 leads to the activation of numerous components of the SA and jasmonic acid signaling pathways, thereby enhancing resistance against a broad spectrum of pathogens^37,38,41^.

Previous investigations indicated that even at relatively high concentrations (e.g., 10 µM), ascr#18 treatment does not suppress plant growth or development across diverse plant species, including major crops (Fig. S1)^35,36^, while providing long-term resistance against viral, bacterial, fungal, and oomycete pathogens^35–37^. This lack of any apparent cost of ascr#18 treatment suggested that ascr#18-enhanced defense responses may be due to priming, motivating the current study. Our results show that ascr#18 treatment primes diverse monocotyledonous and dicotyledonous plants for enhanced defense and trigger chromatin remodeling in regions of defense gene promoters in *Arabidopsis*, resulting in increased disease resistance against a wide range of pathogens both under greenhouse and field conditions.

## Results

### Ascr#18 primes plant cells for enhanced defense

To investigate whether ascr#18 primes plant cells for enhanced defenses, we employed a well-established assay that measures the increase of furanocoumarin secretion by cultured parsley cells induced by the oomycete-derived oligopeptide MAMP, Pep13, a known elicitor of plant defense responses^42,43^. Over the past three decades, this assay has been instrumental in studying defense priming and identifying priming-inducing molecules^44–47^. To test for defense priming by ascr#18, aliquots of culture cells were treated with a range of ascr#18 concentrations or 200 µM SA to determine their capacity to induce defense priming (Fig. 1b). As a known and potent inducer of plant defense priming, SA served as a positive control. Whereas ascr#18 treatment itself did not induce furanocoumarin secretion, pretreatment with ascr#18 for 24 h primed the cells in a dose-dependent manner for enhanced secretion of furanocoumarins induced by adding 50 pM Pep13. Increased furanocoumarin secretion was observed with ascr#18 concentrations as low as 3.75 nM and reached an apparent maximum at 15 nM. Notably, the priming effect of 15 nM ascr#18 was almost as strong as that of a more than 10,000-fold higher concentration of SA (200 µM; Fig. 1b).

### Ascr#18 primes *Arabidopsis* plants for enhanced defense gene expression

Based on the finding that ascr#18 treatment increased accumulation of furanocoumarin in parsley, we hypothesized that ascr#18 may prime defense responses also in other plant species. To test this, we investigated whether pretreatment with ascr#18 followed by infection with *Pseudomonas syringae* pv. tomato (*Pst* DC3000) affects defense priming in *Arabidopsis* (Fig. 1c). Five-week-old *Arabidopsis* plants were treated by root drenching with ascr#18 or control solution. A subset of ascr#18- and mock-treated plants were then inoculated with *Pst* DC3000 or mock solution 24 h later. Six hours after inoculation, leaves were harvested and analyzed for relative transcript abundance of immune genes *PR1, FRK1, WRKY6, WRKY29*, and *WRKY53* using RT-qPCR^20,23,48^. *PR1* encodes a sterol-binding protein that provides protection against oomycete pathogens and acts as a precursor for the immunomodulatory cytokine *CAPE9*^49^. *PR1* is often considered the prototypical marker gene for defense priming^48^ during induced disease resistance in plants^50^. *FRK1* codes for a receptor-like kinase induced by the bacterial MAMP flg22 and is involved in early defense signaling and activation of innate immunity in *Arabidopsis*^50^. *FRK1* is often used as a readout gene for defense priming^48^. The three *WRKY* genes encode defense-related transcription factors and are commonly used as readout genes for defense priming^2,48,51^. Consistent with earlier studies^52–54^, transcript abundance of immune genes was significantly increased 6 h after inoculation with *Pst* DC3000 (Fig. 1d), whereas ascr#18 treatment alone did not significantly activate systemic immune gene expression at this time point. However, immune gene activation following *Pst* DC3000 inoculation was 2–6-fold stronger in plants pretreated with ascr#18 (Fig. 1d). These results show that ascr#18 pretreatment primes *Arabidopsis* plants for enhanced defense gene expression upon pathogen attack, likely leading to increased disease resistance.

### Ascr#18 primes *Arabidopsis* seeds

Because defense priming can have long-lasting effects^55–58^, we next investigated whether treating *Arabidopsis* seeds with ascr#18 would prime defense responses of mature plants (Fig. 1e). To test this, *Arabidopsis* seeds were treated for 24 h by soaking with 1 µM aqueous ascr#18 or mock solution. Treated seeds were extensively washed before planting. Five weeks after planting, plants grown from ascr#18- and mock-treated seeds were either inoculated with *Pst* DC3000 or left uninoculated. Six hours post-inoculation, leaves were harvested and assayed for expression of immune genes. Whereas expression levels of these genes in uninoculated plants did not differ significantly between plants derived from ascr#18- and mock-treated seeds, immune gene expression was significantly induced in *Pst* DC3000*-*inoculated plants (Fig. 1f). Importantly, induction of immune gene expression was significantly higher in plants grown from ascr#18-treated seeds compared to plants grown from mock-treated seeds when inoculated with *Pst* DC3000, demonstrating that seed treatment with low micromolar concentrations of ascr#18 confers defense priming in mature plants.

### Seed priming with ascr#18 provides long-term disease resistance

**Fig. 2:**
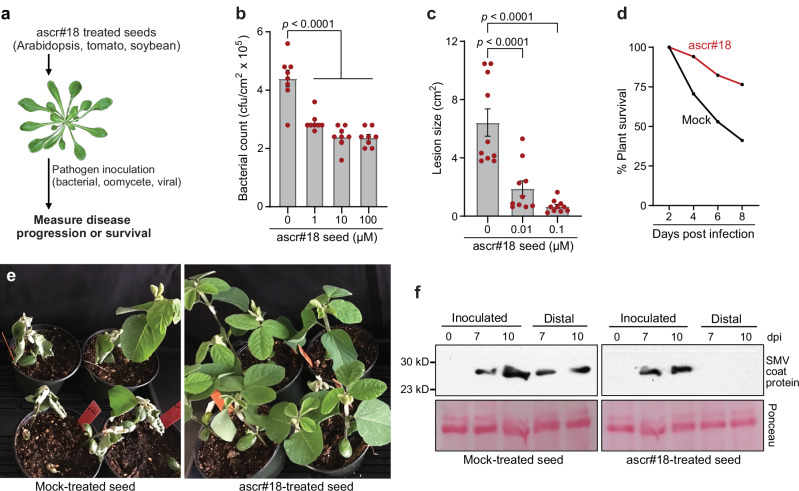
Ascr#18-mediated defense priming enhances resistance to bacterial, oomycete, and viral pathogens. **a** Experimental design for testing for long-term effects of ascr#18 seed treatment on plant defense responses. **b** Enhanced resistance to *Pst* DC3000 in 5-week-old *Arabidopsis* (Col-0) plants grown from ascr#18- or mock-treated seeds. Bacterial multiplication was assayed at 3 dpi. Data are mean ± SEM (*n* = 8). **c** Enhanced resistance to the oomycete *Phytophthora infestans* (pathovar US22) in leaves of 5-week-old tomato (cv. M82) plants grown from ascr#18- or mock-treated seeds. Lesion size was determined at 5 dpi to assess disease symptoms. Data are mean ± SEM (*n* = 10). **d** Soybean plants grown from 100 µM ascr#18- or mock-treated seeds were inoculated with *P. sojae* at the V1 stage. Plant survival was assessed at 2, 4, 6, and 8 dpi (*n* = 15 plants for each condition). Adjusted *p-*values were calculated using ordinary one-way ANOVA and Tukey multiple comparisons post-hoc test. **e** Representative image comparing disease severity 10 days after inoculation with *P. sojae* of soybean plants grown from ascr#18- or mock-treated seeds. Plants were grown in 4-inch diameter pots. **f** Soybean plants grown from 100 µM ascr#18- or mock-treated seeds were inoculated with soybean mosaic virus (SMV) at the V1 stage. SMV accumulation was measured in inoculated and distal systemic leaves at 0, 7, and 10 dpi for inoculated and 7- and 10-dpi for distal using immunoblot analysis with SMV coat protein-specific antibodies. Ponceau staining was used as a control for protein levels (*n* = 2). dpi days post-inoculation. See Fig. S2c for uncropped blot images.

Given that seed treatment with ascr#18 primed immune gene expression in *Arabidopsis*, we asked next whether seed treatment would also confer long-term protection against pathogens (Fig. 2a). We first assessed disease progression in 5-week-old *Pst* DC3000-inoculated *Arabidopsis* plants derived from seeds treated with a range of different ascr#18 concentrations. Bacteria levels in inoculated plants were determined 3 days post-inoculation (dpi). Compared to plants grown from mock-treated seeds, *Pst* DC3000 multiplication was reduced in plants derived from ascr#18-treated seeds at all concentrations tested (Fig. 2b).

Next, we tested the effects of ascr#18 seed treatment in five additional plant-pathogen systems under greenhouse conditions. Tomato plants (cultivar M82) grown from ascr#18-treated seed were challenged with the oomycete pathogen *Phytophthora infestans* 5 weeks after planting. Plants derived from ascr#18-treated seeds showed significantly reduced lesion sizes compared to those grown from mock-treated seeds (Fig. 2c). A similar level of protection was observed in soybeans. Plants grown from mock- or ascr#18-treated seeds were inoculated with *Phytophthora sojae*. Approximately 85% of plants grown from ascr#18-treated seeds survived at 6–8 dpi, compared to only 50% survival of plants grown from mock-treated seeds (Fig. 2d). Disease symptoms at 10 dpi are shown in Figs. 2e and S2. Moreover, seed treatment with ascr#18 also conferred significant protection against soybean mosaic virus (SMV). Virus levels were determined by measuring the amount of virus coat protein in inoculated leaves and uninoculated distal leaves at 7 and 10 dpi. Compared to plants grown from mock-treated seeds, seed treatment with ascr#18 modestly reduced virus levels in inoculated leaves and almost completely blocked virus accumulation in the distal leaves (Fig. 2f).

Seed treatment with ascr#18 also protected monocot plants from disease. Wheat grown from seeds treated with 0.1 or 1.0 μM ascr#18 was challenged by the fungal pathogen *Rhizoctonia solani* 2 days post-planting. Assessment at 12 dpi showed that ascr#18 suppressed fungal replication by 80–90% compared to mock-treated controls (Fig. S3a). Disease symptoms of seedlings at 4 dpi are presented in Fig. S3b. Similarly, rice plants grown from seeds treated with 0, 1.0, or 10 μM of ascr#18 were inoculated with the bacterial pathogen *Xanthomonas oryzae* pv. *oryzae*. Treatment with 10 μM ascr#18 provided significant protection by reducing the length of the disease lesion (Fig. S3c).

Lastly, we asked whether long-term disease protection by ascr#18 could be due to any direct effects of ascr#18 on microbial pathogens. Although previous studies have shown that ascr#18 is rapidly metabolized in plants and becomes undetectable after one to two days post treatment^38,59^, we wondered whether residual ascr#18 could affect pathogen growth and multiplication. To test this, cultures of *Pst* DC3000 and the fungal pathogen *Phakopsora pachyrhizi* were treated with a range of ascr#18 concentrations. We found that ascr#18 treatment did not affect *Pst* DC3000 multiplication or fungal spore germination (Fig. S4). Taken together, our results show that seed treatment with micromolar concentrations of ascr#18 provides significant long-term protection of both monocots and dicots against a diverse range of microbial pathogens.

### The LRR-receptor-like kinase NILR1 is necessary for ascr#18-mediated priming

**Fig. 3:**
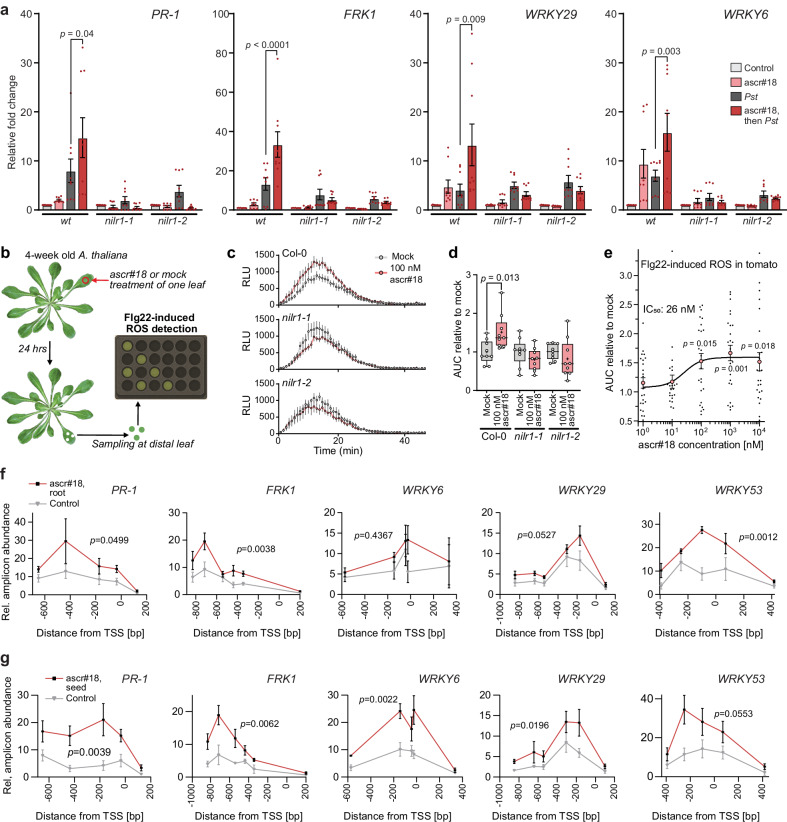
Ascr#18 priming requires the LRR-RLK NILR1 and involves the opening of chromatin at promoter regions of defense genes. **a** *Arabidopsis* (Col-0), *nilr1-1*, or *nilr1-2* plants were root-drenched with 1 µM ascr#18 or control solution for 24 h, before inoculation with *Pst* DC3000, and followed by harvesting leaves 6 h post inoculation. mRNA transcript abundance for *PR1*, *FRK1*, *WRKY29*, and *WRKY6* in the leaves was determined by RT-qPCR and normalized to *β*-tubulin. Data are mean ± SEM (*n* ≥ 8). Experiments were independently repeated 3 times with similar results. Adjusted *p*-values were calculated using one-way ANOVA. **b** Scheme for priming of 4-week-old *Arabidopsis* wildtype plants (Col-0) with 5 µL of mock or ascr#18 (100 nM) via leaf treatment. Twenty-four hours after treatment of a single leaf, leaf discs were collected from a distal (untreated) leaf and incubated with flg22, followed by ROS measurement. **c** ROS response in *Arabidopsis* Col-0, *nilr1-1*, and *nilr1-2* genetic backgrounds. Traces represent the average for 9 plants across 3 independent experiments. Vertical lines represent the standard error of the mean. RLU relative light units. **d** Area under the curve was calculated for the ROS response in individual plants shown in (**c**) and normalized to the respective mocks. Dots represent individual biological repeats. Data are mean ± SEM (*n* = 9). *p*-value determined with an unpaired two-tailed t-test. **e** Dose-response of ascr#18-enhanced ROS induced by flg22 in tomato (M-82). Data points represent the area under the curve (AUC) of the ROS response in individual plants treated with 20 µL of mock or ascr#18 solutions. The data is normalized to the respective mock controls and averaged across 6 independent experiments. Data are mean ± SEM (*n* = 26). Curve was fitted and IC_50_ calculated using a sigmoidal 4-parameter logistic model. Statistical significance was determined using ordinary one-way ANOVA and Dunnett’s multiple comparison test. **f** Roots of 5-week-old *Arabidopsis* plants were drenched in 1 µM ascr#18 or a control solution (control) for 24 h before leaves were harvested and subjected to FAIRE-qPCR at various positions in the promoter and coding sequences of *WRKY6*, *WRKY29*, *WRKY53*, *PR1*, and *FRK1*. **g** *Arabidopsis* seeds were incubated in 1 µM ascr#18 or control solution for 24 h before planting. Leaves from the resulting 5-week-old plants were harvested and subjected to FAIRE-qPCR analysis as in (**f**). In **f**, **g** the data were normalized to *ACTIN2* and represent mean ± SEM (*n* = 3), from 3 independent experiments. *p*-values were calculated as described in “Materials and Methods”. TSS transcriptional start site, bp base pairs. Source data are provided as a Source data file.

Huang et al. recently identified NILR1 as the receptor of ascr#18 in *Arabidopsis*^39^. NILR1 directly binds ascr#18, and two different mutants, *nilr1-1* and *nilr1-2*, were shown to be defective in ascr#18-mediated induction of the priming-marker gene *PR1* and the priming-associated hyper-expression of the readout genes *FRK1*, *WRKY29*, and *WRKY6*^50^. To test whether NILR1 is also required for ascr#18-mediated immune gene priming, we compared expression levels of these defense-related genes under priming conditions in *nilr1-1* and *nilr1-2* mutants with wild type (Col-0). Five-week-old plants of the three genotypes were pretreated via root application with 1 µM ascr#18 for 24 h and then inoculated with *Pst* DC3000. Leaves were harvested at 6 h after inoculation to measure gene expression. Whereas pretreatment with ascr#18 significantly boosted defense gene expression levels in wild-type plants challenged with *Pst* DC3000, no such induction was observed in the two *nilr1* mutants (Fig. 3a), indicating that NILR1 is required for ascr#18-mediated defense priming.

To further validate the role of NILR1 for ascr#18-induced priming of plant defense responses, we asked whether pre-treatment with ascr#18 affects the generation of reactive oxygen species (ROS) in response to the bacterial elicitor flg22^60,61^. ROS bursts are one of the hallmarks of MAMP-triggered immunity, and flg22 is known to robustly induce ROS in *Arabidopsis*^62^. Importantly, this response has been shown to be unperturbed in *Arabidopsis nilr1* mutants^63^. To compare ROS induction by flg22 in ascr#18-pretreated wildtype, *nilr1-1*, and *nilr1-2* plants, single leaves of 4–5-week-old plants were spot-treated with either mock or 100 nM ascr#18 solution (Fig. 3b). Twenty-four hours later, leaf discs were collected from a distal (untreated) leaf, exposed to a luminol-based reaction mix containing flg22 as a ROS elicitor, and the resulting luminescence was measured. We observed an ascr#18-dependent increase in the ROS response in wildtype plants (Fig. 3c, d), similar to the response observed previously with the defense priming agent benzo(1,2,3)thiadiazole-7-carbothioic acid S-methyl ester (BTH) in *Arabidopsis*^31^, indicating that ascr#18-treatment systemically primes ROS responses to flg22. In contrast, flg22-induced ROS responses in ascr#18-treated *nilr1-1* and *nilr1-2* plants were not increased compared to mock-treated plants (Fig. 3c, d), indicating that the enhanced ROS response observed in wildtype requires NILR1, consistent with the result for ascr#18-dependent immune gene priming.

Interestingly, analysis of previously published data for priming of systemic *Arabidopsis* leaves by local *Pseudomonas syringae* pv. *maculicola* (*Psm*) infection revealed chromatin opening in the *NILR1* promoter, accompanied by increased *NILR1* expression^20^ (Supplementary Table S1). This expression does not further increase upon subsequent systemic challenge. Thus, *NILR1* is a target gene of *Psm*-induced defense priming, in addition to being required for ascr#18-induced priming. Increased *NILR1* expression following *Psm*-induced defense priming parallels increased expression of MAMP receptors flagellin sensing 2 (FLS2) and chitin elicitor receptor kinase (CERK1) in response to activation of SA signaling^31^.

Lastly, to demonstrate that the ROS response is not limited to *Arabidopsis*, we tested ascr#18 priming of flg22-induced ROS responses in tomato using the same assay design. (Fig. 3b). We found that, as in *Arabidopsis*, ascr#18 treatment of a single tomato leaf suffices to trigger systemic priming of flg22-induced ROS (Fig. 3e). Together, these results indicate that ascr#18-mediated priming is not restricted to transcriptional responses but also sensitizes early pattern-triggered immune signaling, as reflected by the enhanced flg22-induced ROS burst. This supports the idea that perception of nematode-derived ascarosides prepares plants for faster activation of multiple defense layers upon subsequent pathogen challenge.

### Ascr#18 treatment induces chromatin opening in promoters of defense genes

The finding that ascr#18 treatment results in long-lasting priming of immune genes and increased pathogen resistance suggested potential involvement of chromatin remodeling^23–25,64^. In *Arabidopsis*, defense priming has been shown to be associated with chromatin modifications in the promoters of several immune genes, including *WRKY6*, *WRKY29*, and *WRKY53*^23^. Priming-associated chromatin modifications are manifold and include changes in the methylation status of DNA and alterations in the methylation and acetylation status of specific lysine residues at the amino termini of histones H3 and H4, which reduces the interaction of nucleosome neighbors and lessens ionic DNA-histone interaction, thereby providing docking sites for regulatory proteins^23,64^. Interactions of these regulatory proteins with DNA can lead to localized nucleosome displacement, resulting in nucleosome-free DNA, i.e., open chromatin, that is more accessible to the transcription machinery^20,26^. This facilitates upregulation of gene expression, e.g., in response to signals downstream of pathogen detection^20,25,64^.

To determine whether chromatin modification may underlie priming of immune genes by ascr#18 in *Arabidopsis*, we investigated the effect of ascr#18 root or seed treatment on chromatin in the promoter region of several defense genes. In one set of experiments roots of 5-week-old plants were drenched in 1 µM ascr#18 or a mock solution (control) for 24 h before leaf tissue was harvested and analyzed (Fig. 3f). In another set of experiments seeds were soaked in 1 µM ascr#18 or a mock solution for 24 h before planting. Five weeks later, leaf tissue was harvested and analyzed (Fig. 3g). In both sets of experiments, the harvested leaf tissue was subjected to formaldehyde-assisted isolation of regulatory DNA elements (FAIRE)^26,65^. FAIRE has been shown to accurately identify nucleosome-free sites of DNA corresponding to open chromatin^26,65,66^. As shown in Fig. 3f, root treatment with ascr#18 led to an increase in open chromatin in the promoter regions of *WRKY6, WRKY29, WRKY53, PR1*, and *FRK1* compared to the control group. Similar results were obtained for 5-week-old plants grown from ascr#18- or mock-treated seeds (Fig. 3g). The FAIRE analyses were done along with the gene expression studies reported in Fig. 1c–f, demonstrating that chromatin opening in the promoter regions of these immune genes was associated with only a significant increase in their expression levels in the absence of infection. Instead, ascr#18 treatment resulted in priming for their robust activation following the *Pst* DC3000 challenge.

### Ascr#18 provides long-term disease protection in the field

**Fig. 4:**
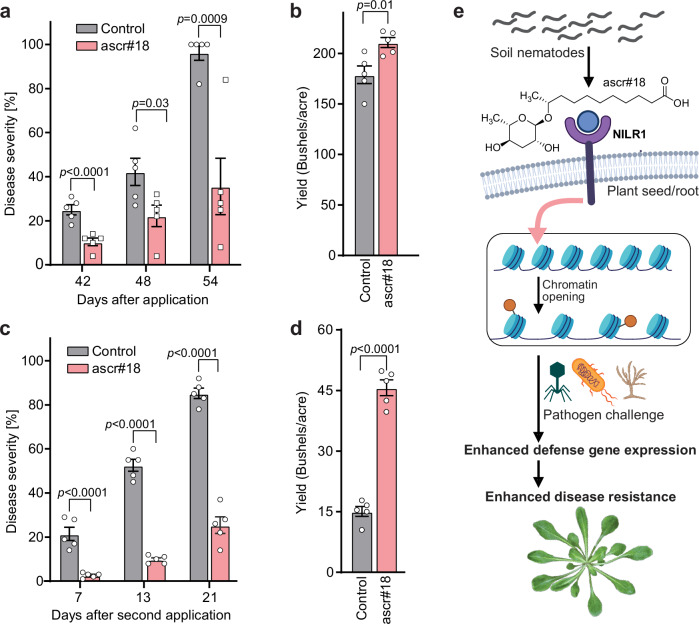
Ascr#18 treatment provides long-term disease resistance under field conditions, and a proposed model for ascr#18-mediated defense priming in plants. **a** Effect of ascr#18 foliar application in corn against natural infection by southern corn leaf blight caused by *Bipolaris maydis*. Ascr#18 (25 mg/acre) was applied at the VT growth stage, and disease severity was scored 42, 48, and 54 days after application. **b** Corn yield from the field experiment in (**a**). Test plots measured 10 feet by 30 feet. Data are mean ± SEM (*n* = 5), where *n* indicates the number of plots, each consisting of four rows. **c** Effect of ascr#18 foliar application in soybean on infection by Asian soybean rust caused by *Phakopsora pachyrhizi*. Test plots were inoculated with the pathogen by introducing infected plant tissue into the soil, and ascr#18 was applied twice at 50 mg/acre during the R1 and R3 growth stages. Disease severity was scored 7, 13, and 21 days after the second ascr#18 application. **d** Soybean yields from the field experiment in (**c**). Test plots measured 6 feet by 30 feet. Data are mean ± SEM (*n* = 5), where *n* indicates the number of plots, each consisting of four rows. Adjusted *p-*values were calculated using two-tailed *t*-test. **e** Model for priming of plant defenses by ascr#18 via chromatin opening (blue: nucleosomes; orange circles: represents anticipated changes in histone acetylation or methylation).

To determine whether the effects of ascr#18 on immune gene activation and disease resistance observed in the greenhouse translate into disease suppression in the field, we conducted field trials with two major row crops, corn and soybean. In the case of corn, we evaluated the effect of ascr#18 priming on southern corn leaf blight, a disease caused by the fungus *Bipolaris maydis* that thrives in warm-temperate and subtropical growing regions, including the Southeastern U.S., and can cause significant yield losses^67^. Ascr#18 was sprayed at a rate of 25 mg per acre at the vegetative tassel emergence growth stage (VT), and disease severity as a result of natural infection with *B. maydis* was evaluated 42, 48, and 54 days after ascr#18 application. We found that ascr#18 significantly reduced disease severity at all time points examined (Fig. 4a). Additionally, ascr#18 treatment resulted in a significant yield increase (Fig. 4b).

Next, we tested whether foliar application of ascr#18 could improve resistance of soybean against Asian soybean rust (ASR), a devastating disease caused by the fungus *P. pachyrhizi* that is ubiquitous in the major soybean growing areas of Latin America and the US and can result in yield losses of 40–80%^68,69^. Soybean fields were inoculated by spreading soybean leaf tissues infected with ASR in the soil during the early stages of growth, specifically between the third and fifth leaf stages (V3–V5). Ascr#18 was applied twice at a rate of 50 mg per acre during the flowering (R1) and pod-development (R3) growth stages, and disease severity was assessed 7, 13, and 21 days after the second application. Compared to untreated controls, ascr#18 significantly reduced disease severity (Fig. 4c) and improved yield (Fig. 4d). These results show that application of ascr#18 at extremely low application rates of 25–50 mg per acre can starkly reduce the severity of major crop diseases and prevent associated yield loss.

## Discussion

Chemical signaling plays a central role in plant ecology and profoundly impacts agricultural and environmental sustainability. The associated interaction networks rely on a vast array of small and large molecules derived from plants, arthropods, nematodes, and diverse microbiota. Importantly, many organisms have acquired the ability to “listen in” on small molecule signals produced by other phyla^70^. For example, fatty acid-glutamine conjugates in insect saliva play a central role in activation of plant defense responses to insect herbivory^71^ analogous to recognition of macromolecular MAMPs, such as flagellin, lipopolysaccharides, peptidoglycan, or chitin derived from bacterial and fungal pathogens^7,72^. Long-term priming of plant defense responses has been demonstrated for a subset of pathogen-derived metabolites, although perception mechanisms and the level of conservation across different plant species have remained unclear^1,2^.

Our demonstration of defense priming by nematode-derived ascarosides provides a striking example for plant recognition of a highly conserved small molecule. Ascaroside biosynthesis and secretion is conserved across free-living and diverse plant- and animal-parasitic nematode species, where the secreted ascarosides principally function as pheromones, coordinating a wide range of behaviors. Like many other pheromones, ascarosides regulate nematode behavior at low concentrations, as low as nano- and picomolar levels. We and others have previously shown that ascarosides affect plant immunity at similarly low concentrations^35–39^. In the current study, we show that even transient exposure of seeds or roots to low micromolar or nanomolar ascr#18 concentrations results in long-term priming of defense responses in diverse dicotyledonous and monocotyledonous plants and provides long-lasting disease protection. However, the optimal concentration of ascr#18 required to activate defense responses varies between crops, as we have demonstrated previously^35–37^. Pretreatment of *Arabidopsis* roots or seeds with ascr#18 enhances the expression of a subset of immune genes upon pathogen challenge. Notably, some immune genes exhibit modest direct induction by ascr#18, followed by a further increase in expression after pathogen challenge. Growth conditions can affect whether these genes exhibit only priming or both direct induction and priming^73^. *WRKY6* and *WRKY29* illustrate this well; in the experiment shown in Fig. 1, both genes are only primed, whereas in Fig. 3, both are directly induced and primed.

Moreover, root or seed treatment of *Arabidopsis*, tomato, soybean, rice, and wheat results in significantly increased resistance to bacterial, oomycete, fungal, and viral pathogens. These results suggest that ascr#18 seed treatment and the resulting long-term priming of plant defenses may represent an effective strategy for improving crop resilience and reducing reliance on chemical pesticides. Whereas other plant defense activators, such as acibenzolar-S-methyl, directly induce defense responses which reduces crop yields^74^, ascr#18 primes plant defenses instead of directly activating them, thereby avoiding fitness and yield penalties.

Using the model plant *Arabidopsis*, we then demonstrate that ascr#18 treatment of roots and seeds results in opening of chromatin in regions of defense gene promoters, likely facilitating enhanced transcriptional responsiveness (Fig. 3f, g). While the precise mechanism underlying increased chromatin accessibility remains unclear, the observed chromatin opening suggests dislodgement of nucleosomes from immune gene regulatory elements, thereby facilitating entry of transcription regulatory proteins that prime immune gene transcription^1,20,75^ (Fig. 4e).

In addition to transcriptional priming of immune genes, we observed enhanced flg22-induced ROS production following ascr#18 treatment. This suggests that ascaroside perception sensitizes early pattern-triggered immune signaling pathways in a NILR1-dependent manner. Because ROS production represents one of the earliest outputs of PRR activation and has previously been associated with chemically induced priming, the enhanced ROS responsiveness observed here supports the idea that ascr#18 primes plant immunity at multiple regulatory levels, ranging from chromatin accessibility and transcriptional competence to early signaling events downstream of immune receptor activation.

Our findings—that treatment of *Arabidopsis* with ascr#18 opens chromatin in the promoter regions of immune genes (Fig. 3f, g), that treatment of *Arabidopsis* seeds with ascr#18 primes defense responses in mature plants (Fig. 1f), and that ascr#18 provides long-term disease resistance in the field (Fig. 4a–d) are consistent with an epigenetic component underlying the long-lasting nature of ascr#18-induced disease resistance. The ascr#18-*Arabidopsis* system, therefore, serves as a valuable model for studying epigenetic immune memory in plants.

Regarding the nature of the epigenetic signal driving somatic transmission of priming, DNA methylation changes, which have been associated with transgenerational priming and disease resistance^76^ are unlikely to be the primary mechanism, given the lack of significant methylation in the promoter regions of the primed defense genes^77^. Therefore, alternative chromatin-based regulatory mechanisms may be involved, including histone replacements or modifications, such as H2A.Z enrichment, histone H3K4 methylation, and H3K27me3. These chromatin marks have been linked to transcriptional responsiveness and memory effects in plant immunity^76^, making them plausible candidates for contributing to ascr#18-induced immune priming.

We further showed that ascaroside priming in *Arabidopsis* requires the recently identified ascr#18 receptor *NILR1* (Fig. 3a–e), a dedicated LRR-RLK that has orthologues in a wide range of dicotyledonous as well as monocotyledonous species^39,63^, and recently it was demonstrated that ascr#18 is also perceived by the potato NILR1 ortholog^78^. Thus, it seems likely that priming with ascr#18 in other plants requires the corresponding *NILR1* orthologs. The requirement of NILR1 for ascr#18-mediated priming is consistent with a role of NILR1 upstream in signal perception and early immune activation, enabling downstream primed responses. In addition, we cannot rule out contributions of other PRRs in ascr#18-mediated activation and priming of the defense response. Nonetheless, broad conservation of NILR1 in plants^39,63,78^ suggests that defense priming by nematode-derived ascaroside pheromones released into the rhizosphere is a broadly conserved and likely ancient feature of plant ecology.

Our results beg the question whether other types of conserved small molecule signals can similarly prime plant defense responses. Interestingly, in *Arabidopsis N-*acyl homoserine lactones (HSLs), a conserved class of bacterial quorum sensing signals, prime cell wall-associated defense that enhance immunity to pathogenic bacteria^79^; however, it is unclear how *Arabidopsis* perceives HSLs and to what extent defense priming by HSLs is conserved in other plants. Like many other MAMPs, ascarosides also appear to be perceived by the mammalian immune system, given that transient exposure to an ascaroside associated with animal-parasitic nematodes has lasting effects on immune responses in mice^40^. Taken together, these findings may suggest that exposure to conserved small molecule signatures from a wide range of micro- and macrobiotic sources plays a significant role in shaping the immune systems of plants as well as animals, including humans.

Reduced biodiversity associated with intense agriculture and the repeated application of a wide range of synthetic chemicals for plant protection has dramatic effects on soil microbiomes, including free-living nematodes^80–84^. Lack of plant defense priming by ascarosides produced by free-living nematodes in the soil could thus contribute to increased disease susceptibility. Our field trials with corn and soybean (Fig. 4a–d) demonstrate that ascr#18 application at very low rates (25–50 mg/acre) significantly reduces the severity of fungal diseases and enhances yield. These results highlight the potential of harnessing an in-depth understanding of plant-biotic interactions to develop sustainable and environmentally friendly approaches for disease management in agriculture.

## Methods

### Ascaroside treatment solutions

The ascr#18, synthesized using a method described previously^85^, was dissolved in ethanol to prepare millimolar stock solutions that were stored at −20 °C. Aqueous ascr#18 solutions were prepared freshly on the day of the experiment by diluting the stock solution into water or media. Control treatment solutions were prepared to contain equal amounts of ethanol. The final ethanol concentration of all solutions was 0.1% or less.

### Cultivation of parsley suspension cells and its use in priming experiments

The cultivation of parsley cells was done analogous to a previous report^22^. For inoculation, 35 mL of a 1-week-old parsley (*Petroselinum crispum*) cell suspension culture was transferred to 500-mL Erlenmeyer flasks containing 120 mL of sterile Gamborg’s B5 medium (3.16 g/L medium powder including vitamins, 20 g/L sucrose, 2 mg/L 2,4-dichlorophenoxyacetic acid, and 0.25 g/L magnesium sulfate; pH 5.5). The culture was incubated on a shaker at 120 rpm at 24 °C in the dark. Three days after subculturing, 1 mL of the cell suspension was transferred to each well of a 24-well CELLSTAR microplate (Greiner Bio-One). Cells were then treated with 200 µM of SA (as a positive control) and different concentrations of ascr#18. After 24 h of incubation, 1 mL aliquot of cultured parsley cells were treated with 0.05 nM of custom-synthesized Pep13 defense elicitor (^+^H_3_N-VWNQPVRGFKVYE-COO)^86^. Twenty-four hours after Pep-13 addition, the fluorescence of furanocoumarins in individual wells of the microplates were measured using a CLARIOstar multi-mode microplate reader (BGM LABTECH, Germany) at 335 nm excitation and 398 nm emission.

### Cultivation of *Pseudomonas syringae* DC 3000 for plant inoculation

*Pst* DC3000 was grown in petri dishes containing King’s B medium^87^ supplemented with 100 µg/mL rifampicin, 25 µg/mL kanamycin, and 10 g/L agar. After incubation for 2 days at 28 °C, 4–5 colonies were transferred to a 250-mL Erlenmeyer flask containing 50 mL King’s B medium containing 100 µg/mL rifampicin and 25 µg/mL kanamycin. The flask was incubated overnight at 28 °C on a rotary shaker (at 220 rpm) before the bacterial culture was harvested, transferred to a 50-mL plastic tube, and centrifuged at 1800 × *g* at 16 °C for 8 min. The supernatant was discarded, and the pellet was suspended in 50 mL of 10 mM MgCl_2_. After another centrifugation at 1800 × *g* at 16 °C for 8 min, the pellet was suspended in 50 mL of 10 mM MgCl_2_. One mL of bacterial suspension was diluted with 10 mM MgCl_2_ to an OD_600_ of 0.0002, resulting in ~3 × 10^8^  cfu/mL.

### *Arabidopsis* growth conditions

Unless otherwise stated, *Arabidopsis thaliana* wildtype (ecotype Col-0) was grown in a growth chamber at 22 °C using a 16 h light/8 h dark cycle and 70% humidity.

### Root, seed, and spray treatments of *Arabidopsis*

For *Arabidopsis* root treatment, 5-week-old plants were watered with 30 mL of an aqueous solution containing 1 µM ascr#18 or 0.1% ethanol (control). For *Arabidopsis* seed treatment, seeds were treated by incubating in 2 mL test tubes containing an aqueous solution of 1, 10, or 100 µM of ascr#18 in 0.1% ethanol or 0.1% ethanol (control). Seeds were soaked in test tubes while shaking at room temperature in the dark for 24 h. After washing three times with deionized water, the seeds were planted on soil and grown as described above. Twenty-four hours after root treatment or 5 weeks after planting of the treated seeds, plants were analyzed (i) for immune gene expression following pathogen challenge (Fig. 1), (ii) for enhanced long-term resistance (Fig. 2), or (iii) for the state of chromatin in the promoter of immune genes (Fig. 3). For sterile growth conditions, *Arabidopsis* seeds were surface sterilized by soaking in a 50% bleach solution for 10 min and washed extensively with sterilized water before planting on *Arabidopsis* growth media containing 2.15 g/L Murashige and Skoog salts (Sigma-Aldrich, USA) and 10 g/L sucrose. The pH was adjusted to 6.0 using KOH. For solid growth media, 8 g/L agar (Sigma-Aldrich, USA) was added before autoclaving.

Twelve-day-old *Arabidopsis* seedlings growing on a 0.5X MS agar plate were spray-treated with 1 µM of ascr#18 in 0.1% ethanol or 0.1% ethanol solution for 48 h. The seedlings were then transferred to soil and grown under the conditions described under the *Arabidopsis* growth conditions above. Measurement was taken 30 days post-treatment by weighing above-ground biomass (Fig. S1a).

### Direct effect of ascr#18 on growth and multiplication of pathogens

For the direct effect of ascr#18, aliquots of *Phakopsora pachyrhizi* suspension (1 mg/mL) in 0.01% Tween-20 were treated with ethanol (control), ascr#18 (0.1, 1, 10, or 100 µM) in ethanol. After incubation for 3 h at 20 °C, the germination rate of approximately 3 × 100 spores per aliquot was determined relative to untreated spores (Fig. S4a). For *Pst* DC3000, OD_600_ of the overnight culture grown at 28 °C was determined and then diluted to OD_600_ 2, 0.2, 0.002, and 0.0002. Five millilitres of each dilution was treated with ethanol (control) or ascr#18 (10, 100 nM, or 1 µM). One aliquot was left untreated. Cultures were incubated overnight at 28 °C and 220 rpm on a rotary shaker before serial dilution and plating of 5 µL drops on petri dishes containing King’s B medium supplemented with 100 µg/mL rifampicin, 25 µg/mL kanamycin, and 10 g/L agar. After 48 h of incubation at 28 °C, colony-forming units (cfu) were determined (Fig. S4b).

### Immune gene activation/priming and enhanced resistance analyses in *Arabidopsis*

*For immune gene priming and activation:* two leaves of 4–5-week-old plants root drenched with 1 µM ascr#18 or 0.1% ethanol (control) for 24 h or plants grown from seeds treated with 1 µM ascr#18 or 0.1% ethanol (control) were syringe infiltrated with a suspension of virulent *Pst* DC3000 in 10 mM MgCl_2_ at a density of ~1 × 10^5^ cfu/mL or with 10 mM MgCl_2_ only as a control (Fig. 1). Leaves were harvested 6 h after inoculation and subjected to RT-qPCR analysis for the quantification of gene-specific mRNA transcripts. RNA was isolated from leaves using the TRIZOL method^88^. Two microliters of RNA were subjected to DNase digestion followed by cDNA synthesis using RevertAid reverse transcriptase (Thermo Scientific, USA). mRNA transcript abundance was determined by RT-qPCR on C1000 TouchTM Thermal Cycler (CFX 284TM Real-Time System, Bio-Rad, Germany) using gene-specific primers (Integrated DNA Technology, USA; Thermo Fisher, Germany; see Supplementary Table S1) and iTaqTM SYBR® Green Supermix (Bio-Rad, Germany). Data were normalized to the level of mRNA transcript of *ACTIN2 (*AT3G18780), using the 2(-delta delta C(T)) method^89^.

*For pathogen analysis*: three rosette leaves of 4–5-week-old plants grown from seeds treated with increasing concentrations of ascr#18 or ethanol (control) were syringe infiltrated with a suspension of virulent *Pst* DC3000 in 10 mM MgCl_2_ at a density of ~1 × 10^5^ cfu/mL or with 10 mM MgCl_2_ only as a control (Fig. 2). Bacterial count was done 3 dpi as described in Tian et al.^90^. Briefly, 3 leaf discs with a diameter of 0.7 cm were collected from 3 plants and placed into a single tube, serving as one replicate. Serial dilutions were performed after bacteria recovery using 1 mL of 10 mM MgCl_2_. For this, 20 μl of tissue extract from each tube was added to the wells of a microtiter plate containing 180 μl of 10 mM MgCl_2_, and serial 10-fold dilutions were prepared with a multi-channel pipette. Drops of 5 μl from each dilution were spotted onto a 150-mm petri dish of Luria-Bertani (LB) containing rifampicin, and the plates were incubated at 28 °C. Bacterial counts were performed 48 h after incubation.

### Tomato-pathogen assay

Tomato (*Solanum lycopersicon)* cultivar M82 seeds were placed in 50 mL conical centrifuge tubes and incubated with water containing 0.1% ethanol (control) or solutions of 0.01 or 0.1 of µM of ascr#18 in water containing 0, 1% ethanol. The tubes were placed on a shaker kept at 4 °C in a cold room in the dark for 24 h. Subsequently, seeds were washed three times with water and planted in the soil. The plants were grown in the greenhouse with a 16-h light/8-h dark regime at 22 °C with 70% relative humidity. Five-week-old plants grown from ascr#18- or mock-treated seeds were infected with a virulent strain of *Phytophthora infestans* (US22) using a detached-leaflet assay^91^, in which the abaxial leaflet surface was inoculated by dropping 20 μl of sporangia suspension (4000 sporangia/mL). The sporangia suspension was incubated for 3 h at 4 °C for zoospore release before leaflet inoculation. The inoculated leaflets were kept in petri dishes containing water agar and were incubated at 15 °C. The blighted area was measured at 5 dpi.

### Soybean-pathogen assays

For soybean (*Glycine max* (L.) Merr.) seed treatment, seeds of cultivars Essex or Harosoy were treated by incubating in 50 mL test tubes containing an aqueous solution of 100 µM of ascr#18 in 0.1% ethanol or 0.1% ethanol (control). Seeds were soaked in test tubes while shaking at room temperature in the dark for 6 h. After washing three times with deionized water, the seeds were planted on soil. The plants were grown in the greenhouse with 25 and 20 °C day and night temperatures, respectively. Soybean plants at the V1 stage were inoculated with the pathogens as follows:

For the soybean mosaic virus strain G5 (SMV-G5) assay, soybean cultivar Essex was used as the host of SMV. For inoculation, virus-infected plant tissue was homogenized in 0.01 M phosphate buffer, mixed with a small amount of carborundum, and rub-inoculated on leaves of plants grown from treated seeds at the V1 stage. Tissue from the inoculated and uninoculated systemic leaves were collected at different time intervals (0, 7, 10 dpi for inoculated leaves; 7 and 10 dpi for uninoculated/distal leaves) and analyzed by immunoblotting using SMV coat protein-specific antibodies. Rabbit polyclonal antibodies against bacterially expressed coat protein of a Kentucky isolate of SMV G7 were developed by Dr. S. A. Ghabrial^92^. For the *Phytophthora sojae* (races 1 and 3) assay, soybean cultivar Harosoy was used as the host. *P. sojae* was grown on V8 agar at 25 °C in the dark^93^. Soybean inoculations with *P. sojae* was done at the V1 stage^94^. Soybean seedlings were inoculated by placing a small amount of mycelium in a vertical slit wound (approximately 1 cm in length) made 2–3 mm below the first trifoliate leaf, and then covering the inoculated wound with parafilm. Mock inoculations were carried out with agar plugs without mycelia. Disease progression was measured as the number of plants killed in response to *P. sojae* infection.

### Rice-pathogen assays

For rice *(Oryza sativa* cv. Kitaake*)* seed treatment, seeds were incubated in 15 mL test tubes containing 1 or 10 µM of ascr#18 in 0.1% ethanol or 0.1% ethanol (control). Seeds were soaked in test tubes while shaking at room temperature in the dark for 24 h. After washing three times with deionized water, seeds were germinated in Petri dishes for 7 days before transplanting to pots containing 50:50 mixture of Pro-mix potting mix:profile greens grade and grown in a greenhouse at 85% relative humidity with 16 h light/8 h dark and 30 °C day/25 °C night. Three-week-old plants were inoculated with *Xanthomonas oryzae* pv. *oryzae* strain PXO86. For inoculum preparation, cultures of *X. oryzae* were incubated at 28 °C on peptone-sucrose agar medium for 24 h before the bacteria scraped from the plate and were suspended in sterile water to an optical density (OD_600_) of 0.2 (1 × 10^8^ CFU/mL). Two leaves from each plant were inoculated by dipping a scissors into the bacterial suspension before cutting each leaf 4 cm from the tip^95^. For the progression of infection, lesion lengths were measured 14 dpi (Fig. S3c).

### Wheat-*Rhizoctonia* assays

Seeds of wheat (*Triticum aestivum* cv. Kanzler) were treated with 0, 0.1, or 1 µM of ascr#18 in 0.1% ethanol or 0.1% ethanol (control) for 24 h before planting on filter paper. Two-day-old seedlings were inoculated with *Rhizoctonia solani* Kühn inoculum (anastomosis group AG8; teleomorph *Thanatephorus cucumeris* [Frank] Donk^96^) prepared by grounding oat seeds infected with *R. solani*^97^. The fungus was propagated on potato dextrose agar (PDA; Difco, Sparks, NV). Oat kernels were inoculated after 3 weeks of growth at 22–25 °C, oat kernels were evenly covered with mycelia. Controls consisted of autoclaved and dried oats that were not inoculated with the pathogen. Colonized and noncolonized oats were ground in coffee grinders, and propagules were enumerated by plating on a selective medium. Infection strength was measured 12 dpi by quantifying *R. solani internal transcribed spacers 1* (*ITS1*) using DNA extracted from infected root tissues (Fig. S3a, b). Each treatment included 20 seeds, and the data from three independent experiments were averaged^95^. For growth analysis, wheat seeds (cv. Louise) were soaked for 24 h in mock solution or different concentrations of ascr#18 (0.01, 0.1, and 1 µM) before planting in soil. They were grown under greenhouse conditions with a 16-h light/8-h dark (22 °C) regime and 70% relative humidity. Plant growth was assessed by measuring the distance of the second node from the base of the plants 10 days after planting (Fig. S1b).

### FAIRE-qPCR analysis

All the leaves of at least five plants per treatment were root drenched for 24 h with 1 µM ascr#18 (Fig. 3f), or of plants grown from seeds treated for 24 h with 1 µM ascr#18 or control solutions (Fig. 3g) were collected into 120-mL plastic tubes and subjected to FAIRE-qPCR analysis^20^. The tubes were filled to 80 mL with crosslinking buffer (10 mM HEPES (pH 7.8), 400 mM sucrose, 5 mM ß-mercaptoethanol, 0.1 mM PMSF, 3% (v/v) formaldehyde) and subjected to successive rounds of vacuum infiltration for 1.0, 1.5, and 1.0 min. Excessive formaldehyde was quenched by adding freshly prepared glycine to a final concentration of 125 mM followed by vacuum infiltration for 1.0, 1.5, and 1.0 min. Leaves were then transferred to a beaker and thoroughly washed with tap water. After drying by putting leaves between paper towels, the leaves were quickly frozen in liquid nitrogen. Frozen leaves were ground into a fine powder using mortar and pestle. Three aliquots of powder were used per leaf sample and processed concurrently. For each aliquot, two spatula tips of frozen ground leaf powder (~80 mg) were suspended in 850 µL of DNA extraction buffer (100 mM Tris-HCl, 100 mM NaCl, 50 mM EDTA (pH 8.0)). Samples were shaken at 25 °C for 3 min, followed by sonication using Diagenode Bioruptor® (power set at high, 10 times, 30 s on/30 s off). After centrifugation for 5 min at 16,100 × *g* at room temperature, supernatants of the three aliquots of the same sample were pooled in a 50-mL plastic tube. Three 700-µL aliquots of pooled supernatant were transferred to a fresh 1.5-mL tube. 80 µL of the supernatant was mixed with 540 µL of extraction buffer and incubated at 65 °C overnight to reverse formaldehyde crosslinks (input control). The remaining supernatant was kept frozen overnight at −20 °C. The next day, samples were thawed and centrifuged at room temperature for 5 min at 16,100 × *g*. Five hundred fifty microlitres of the supernatant were supplemented with the same volume of phenol-chloroform, mixed and centrifuged at 20,800 × *g* for 15 min at 4 °C. Three hundred fifty microlitres of the aqueous phase was transferred to a fresh tube. After the addition of one volume of chloroform, samples were mixed and centrifuged at 16,100 × *g* as before. DNA in 200 µL of the supernatant was precipitated by addition of two volumes ice-cold 96% ethanol followed by incubation at 20 °C for 30 min. DNA was then collected by centrifugation at 20,800 × *g* for 20 min at 4 °C. The DNA pellet was washed with 70% (v/v) ethanol and dried on the bench for 1 h. The DNA pellet was then suspended in 200 µL deionized H_2_O and incubated at 70 °C for 15 min. Relative abundance of DNA fragments was determined by qPCR using site-specific primers (Supplementary Table S2) relative to input and normalized to the level of a coding sequence of *ACTIN2*. Statistical significance of the difference of the resulting FAIRE-seq curves for samples from untreated vs. ascr#18-treated plants (Fig. 3f, g) was assessed using the modified Chi-squared method introduced by Hristova and Wimley^98^.

### Treatment of *Arabidopsis* and *nilr1* mutants and RT-qPCR

Seeds of *Arabidopsis* wildtype (Col-0) and *nilr1-1* and *nilr1-2* mutants^63^ were germinated and grown in a greenhouse for about 5 weeks, as described above, and then used for root treatment. Briefly, the *Arabidopsis* plants were watered with 30 mL of an aqueous solution containing 0.1% ethanol plus 1 µM ascr#18 or 0.1% ethanol (control). Twenty-four hours later, leaves of the treated plants were syringe infiltrated with a suspension of virulent *Pst* DC3000 in 10 mM MgCl_2_ at a density of ~1 × 10^5^ cfu/ mL or with 10 mM MgCl_2_ (control). Leaves were harvested 6 hours after inoculation and subjected to RT-qPCR analysis to quantify gene-specific mRNA transcripts. mRNA was isolated from leaves using the Dynabeads mRNA DIRECT Kit (Thermo Scientific, USA). DNA contamination was removed by treatment with DNase I (Invitrogen, USA). First-strand cDNA was synthetized from 50 ng of mRNA using ProtoScript II Reverse Transcriptase (NEB, USA). The qRT-PCR assay was carried out in CFX96 Real-Time System (Bio-Rad, USA), and transcripts were amplified using iTaq™ Universal SYBR Green Supermix (Bio-Rad) from 1 µL of 20X diluted cDNA in a total of 20 µL reaction using 1 µL of each 10 µM gene-specific primers (see Supplementary Table S3). All assays consisted of 3 technical replicates for each mRNA sample, using 1 µg of mRNA per sample. PCR was started with an activation and DNA denaturation step (95 °C for 3 min), followed by 40 cycles of 95 °C for 20 s and 60 °C for 40 s. mRNA abundance of the 4 defense genes (*PR-1*, *FRK1*, *WRKY29*, and *WRKY6*) was normalized to that of *Arabidopsis* *β-tubulin* gene (AY059075).

### ROS assays

Luminol-based ROS assays were performed in *Arabidopsis* (Col-0) and tomato (M-82) using a protocol adapted from Bisceglia et al.^99^. *Arabidopsis* or tomato plants were grown in a growth chamber kept at 23 °C, 60% relative humidity, and 16 h light. Four to five-week-old plants were treated on one leaf with 5 µL of aqueous mock or ascr#18 solution at the indicated concentrations. 24 h after treatment, leaf discs (4 mm diameter) were collected from a fully expanded leaf distal to the treated leaf and floated overnight in 100 µL of sterile water in white 96‑well plates (one disc per well) at room temperature in the dark. On the next day, the water was replaced with 100 µL of sterile assay solution containing 40 µM luminol (Sigma-Aldrich 123072), 5 µg mL^−^¹ horseradish peroxidase (Sigma-Aldrich P8125), and 10–50 nM flg22 (PhytoTech P6622). Chemiluminescence was measured immediately as Relative Light Units (RLU) using a BioTek Synergy HTX microplate reader with 0.2 s integration per well at 50 s intervals at room temperature, and data were represented as RLU over time or analyzed as area under the curve (AUC).

### Field testing

Two field trials were conducted in Georgia during the 2021–22 growing season, one each for corn and soybean. The experimental design for all field trials was a randomized complete block design with five replicates (*n* = 5) per treatment. Each plot consisted of four rows, 10 × 30 ft per plot for corn and 6 × 30 ft for soybean. For corn, the Southern leaf corn blight (SCLB)-susceptible cultivar PIO1960RR was planted in May 2022 and was harvested in September 2022. Ascr#18 was applied at 25 mg/acre (1 µM, 20-gallon/acre application rate with 0.25% of a nonionic surfactant, Activator 90 (Loveland)) by spraying at the vegetative tassel (VT) growth stage, and natural infection of SCLB was measured in ascr#18-treated and untreated plants. For soybean, the Asian soybean rust (ASR)-susceptible cultivar PIO95Y70 was planted in June 2022 and harvested in October 2022. Pathogen inoculation was performed by introducing plant tissue infected with ASR into the soil. The ascr#18 was applied twice at 50 mg/acre (2 µM, 20-gallon/acre application rate with 0.25% of a nonionic surfactant, Activator 90 (Loveland)) by spraying at the reproductive stage R1 and R3. Disease severity was assessed visually in the center 2 rows for each plot of corn and soybean. The center 2 rows of each plot were harvested to evaluate the yield impact of ascr#18 treatment. Independent contractor GLC Consulting performed all experiments according to agronomic practices relevant to standard practices in the corn and soybean regions of the US.

### Statistics and reproducibility

All experiments were independently repeated at least twice, at different times, with similar results. All data are expressed as mean ± SEM. Statistical analysis was conducted using two-tailed t-tests or one-way analysis of variance (ANOVA) with Tukey post hoc multiple comparisons test, as indicated in the Figure legends. A *p* < 0.05 was considered significant. The experiments were not randomized (except for field trials), and the investigators were not blinded to allocation during the experiments and outcome assessment. No statistical method was used to predetermine sample size, and no data were excluded from the analyses. Statistical analyses were performed using either Microsoft Excel (Microsoft, Redmond, WA) or GraphPad Prism, version 10 (GraphPad Software, La Jolla, CA).

### Reporting summary

Further information on research design is available in the Nature Portfolio Reporting Summary linked to this article.

## Supplementary information

Transparent Peer Review File

Supplementary Information

Description of Additional Supplementary Files

Supplementary Data 1

Reporting Summary

## Data Availability

Source data for all figures are provided with this paper (see Supplementary Data 1). Data shown in Supplementary Table S1 were deposited at the European Nucleotide Archive (ENA), accession PRJEB32929 (Baum et al. 2019). All other data are available from the corresponding author (or other sources, as applicable) on reasonable request.

## References

[1] Conrath, U., Beckers, G. J., Langenbach, C. J. & Jaskiewicz, M. R. Priming for enhanced defense. *Annu. Rev. Phytopathol.* **53**, 97–119 (2015). 26070330 10.1146/annurev-phyto-080614-120132

[2] Hönig, M., Roeber, V. M., Schmülling, T. & Cortleven, A. Chemical priming of plant defense responses to pathogen attacks. *Front. Plant Sci.* **14**, 1146577 (2023). 37223806 10.3389/fpls.2023.1146577 PMC10200928

[3] Quintin, J. et al. *Candida albicans* infection affords protection against reinfection via functional reprogramming of monocytes. *Cell Host Microbe* **12**, 223–232 (2012). 22901542 10.1016/j.chom.2012.06.006 PMC3864037

[4] Zipfel, C. Pattern-recognition receptors in plant innate immunity. *Curr. Opin. Immunol.* **20**, 10–16 (2008). 18206360 10.1016/j.coi.2007.11.003

[5] Boutrot, F. & Zipfel, C. Function, discovery, and exploitation of plant pattern recognition receptors for broad-spectrum disease resistance. *Annu. Rev. Phytopathol.* **55**, 257–286 (2017). 28617654 10.1146/annurev-phyto-080614-120106

[6] Frost, C. J., Mescher, M. C., Carlson, J. E. & De Moraes, C. M. Plant defense priming against herbivores: getting ready for a different battle. *Plant Physiol.* **146**, 818–824 (2008). 18316635 10.1104/pp.107.113027 PMC2259053

[7] Abdul Malik, N. A., Kumar, I. S. & Nadarajah, K. Elicitor and receptor molecules: orchestrators of plant defense and immunity. *Int. J. Mol. Sci.* **21**, 963 (2020). 10.3390/ijms21030963 PMC7037962 32024003

[8] Ecker, J. R. & Davis, R. W. Plant defense genes are regulated by ethylene. *Proc. Natl. Acad. Sci. USA* **84**, 5202–5206 (1987). 16593860 10.1073/pnas.84.15.5202 PMC298822

[9] Yang, C. et al. Activation of ethylene signaling pathways enhances disease resistance by regulating ROS and phytoalexin production in rice. *Plant J.* **89**, 338–353 (2017). 27701783 10.1111/tpj.13388

[10] Löwe, M. et al. N-hydroxypipecolic acid primes plants for enhanced microbial pattern-induced responses. *Front. Plant Sci.* **14**, 1217771 (2023). 37645466 10.3389/fpls.2023.1217771 PMC10461098

[11] Li, Q. et al. N-hydroxypipecolic acid triggers systemic acquired resistance through extracellular NAD(P). *Nat. Commun.* **14**, 6848 (2023). 37891163 10.1038/s41467-023-42629-0 PMC10611778

[12] Wasternack, C. Jasmonates: an update on biosynthesis, signal transduction and action in plant stress response, growth and development. *Ann. Bot.* **100**, 681–697 (2007). 17513307 10.1093/aob/mcm079 PMC2749622

[13] Huot, B., Yao, J., Montgomery, B. L. & He, S. Y. Growth-defense tradeoffs in plants: a balancing act to optimize fitness. *Mol. Plant* **7**, 1267–1287 (2014). 24777989 10.1093/mp/ssu049 PMC4168297

[14] Kliebenstein, D. J. False idolatry of the mythical growth versus immunity tradeoff in molecular systems plant pathology. *Physiol. Mol. Plant Pathol.* **95**, 55–59 (2016).

[15] Zimmerli, L., Jakab, G., Métraux, J.-P. & Mauch-Mani, B. Potentiation of pathogen-specific defense mechanisms in *Arabidopsis* by β-aminobutyric acid. *Proc. Natl. Acad. Sci. USA* **97**, 12920–12925 (2000). 11058166 10.1073/pnas.230416897 PMC18865

[16] Conrath, U. et al. Priming: getting ready for battle. *Mol. Plant Microbe Interact.* **19**, 1062–1071 (2006). 17022170 10.1094/MPMI-19-1062

[17] Boller, T. & Felix, G. A renaissance of elicitors: perception of microbe-associated molecular patterns and danger signals by pattern-recognition receptors. *Annu. Rev. Plant Biol.* **60**, 379–406 (2009). 19400727 10.1146/annurev.arplant.57.032905.105346

[18] Martinez-Medina, A. et al. Recognizing plant defense priming. *Trends Plant Sci.* **21**, 818–822 (2016). 27507609 10.1016/j.tplants.2016.07.009

[19] van Hulten, M., Pelser, M., van Loon, L. C., Pieterse, C. M. & Ton, J. Costs and benefits of priming for defense in *Arabidopsis*. *Proc. Natl. Acad. Sci. USA* **103**, 5602–5607 (2006). 16565218 10.1073/pnas.0510213103 PMC1459400

[20] Baum, S. et al. Isolation of open chromatin identifies regulators of systemic acquired resistance. *Plant Physiol.* **181**, 817–833 (2019). 31337712 10.1104/pp.19.00673 PMC6776868

[21] Beckers, G. J. & Conrath, U. Priming for stress resistance: from the lab to the field. *Curr. Opin. Plant Biol.* **10**, 425–431 (2007). 17644024 10.1016/j.pbi.2007.06.002

[22] Schillheim, B. et al. Sulforaphane modifies histone H3, unpacks chromatin, and primes defense. *Plant Physiol.* **176**, 2395–2405 (2018). 29288231 10.1104/pp.17.00124 PMC5841731

[23] Jaskiewicz, M., Conrath, U. & Peterhänsel, C. Chromatin modification acts as a memory for systemic acquired resistance in the plant stress response. *EMBO Rep.* **12**, 50–55 (2011). 21132017 10.1038/embor.2010.186 PMC3024125

[24] Kang, H., Fan, T., Wu, J., Zhu, Y. & Shen, W. H. Histone modification and chromatin remodeling in plant response to pathogens. *Front. Plant Sci.* **13**, 986940 (2022). 36262654 10.3389/fpls.2022.986940 PMC9574397

[25] Ding, B. & Wang, G.-L. Chromatin versus pathogens: the function of epigenetics in plant immunity. *Front. Plant Sci.* **6**, 675 (2015). 10.3389/fpls.2015.00675 PMC4557108 26388882

[26] Baum, S., Reimer-Michalski, E. M., Jaskiewicz, M. R. & Conrath, U. Formaldehyde-assisted isolation of regulatory DNA elements from *Arabidopsis* leaves. *Nat. Protoc.* **15**, 713–733 (2020). 32042178 10.1038/s41596-019-0277-9

[27] Zhang, Y. & Reinberg, D. Transcription regulation by histone methylation: interplay between different covalent modifications of the core histone tails. *Genes Dev.* **15**, 2343–2360 (2001). 11562345 10.1101/gad.927301

[28] Bird, A. DNA methylation patterns and epigenetic memory. *Genes Dev.* **16**, 6–21 (2002). 11782440 10.1101/gad.947102

[29] Kim, M. Y. & Zilberman, D. DNA methylation as a system of plant genomic immunity. *Trends Plant Sci.* **19**, 320–326 (2014). 24618094 10.1016/j.tplants.2014.01.014

[30] Eberharter, A. & Becker, P. B. Histone acetylation: a switch between repressive and permissive chromatin. Second in review series on chromatin dynamics. *EMBO Rep.* **3**, 224–229 (2002). 11882541 10.1093/embo-reports/kvf053 PMC1084017

[31] Tateda, C. et al. Salicylic acid regulates *Arabidopsis* microbial pattern receptor kinase levels and signaling. *Plant Cell* **26**, 4171–4187 (2014). 25315322 10.1105/tpc.114.131938 PMC4247590

[32] Srinivasan, J. et al. A blend of small molecules regulates both mating and development in *Caenorhabditis elegans*. *Nature* **454**, 1115–1118 (2008). 18650807 10.1038/nature07168 PMC2774729

[33] von Reuss, S. H. & Schroeder, F. C. Combinatorial chemistry in nematodes: modular assembly of primary metabolism-derived building blocks. *Nat. Prod. Rep.* **32**, 994–1006 (2015). 26059053 10.1039/c5np00042d PMC4884655

[34] Machado, R. A. R. & Von Reuss, S. H. Chemical ecology of nematodes. Chimia **76**, 945–953 (2022). 38069790 10.2533/chimia.2022.945

[35] Klessig, D. F. et al. Nematode ascaroside enhances resistance in a broad spectrum of plant–pathogen systems. *J. Phytopathol.* **167**, 265–272 (2019).

[36] Kamboj, A. et al. The nematode signaling molecule ascr#18 induces prepenetration defenses in wheat against a leaf rust fungus. *J. Plant Dis. Prot.* **131**, 2053–2062 (2024).

[37] Manosalva, P. et al. Conserved nematode signalling molecules elicit plant defenses and pathogen resistance. *Nat. Commun.* **6**, 7795 (2015). 26203561 10.1038/ncomms8795 PMC4525156

[38] Manohar, M. et al. Plant metabolism of nematode pheromones mediates plant-nematode interactions. *Nat. Commun.* **11**, 208 (2020). 31924834 10.1038/s41467-019-14104-2 PMC6954178

[39] Huang, L. et al. NILR1 perceives a nematode ascaroside triggering immune signaling and resistance. *Curr. Biol.* **33**, 3992–3997 (2023). 37643618 10.1016/j.cub.2023.08.017

[40] Shinoda, K. et al. Nematode ascarosides attenuate mammalian type 2 inflammatory responses. *Proc. Natl. Acad. Sci. USA* **119**, e2108686119 (2022). 10.1073/pnas.2108686119 PMC8892368 35210367

[41] Robert-Seilaniantz, A., Grant, M. & Jones, J. D. Hormone crosstalk in plant disease and defense: more than just jasmonate-salicylate antagonism. *Annu. Rev. Phytopathol.* **49**, 317–343 (2011). 21663438 10.1146/annurev-phyto-073009-114447

[42] Thulke, O. & Conrath, U. Salicylic acid has a dual role in the activation of defence-related genes in parsley. *Plant J.* **14**, 35–42 (1998). 15494053 10.1046/j.1365-313X.1998.00093.x

[43] Katz, V. A., Thulke, O. U. & Conrath, U. A benzothiadiazole primes parsley cells for augmented elicitation of defense responses. *Plant Physiol.* **117**, 1333–1339 (1998). 9701589 10.1104/pp.117.4.1333 PMC34897

[44] Schmitz, K., Werner, L. & Conrath, U. High-throughput screening for defense priming-inducing compounds in parsley cell cultures. *Bio Protoc.* **11**, e4200 (2021). 34761072 10.21769/BioProtoc.4200 PMC8554801

[45] Hoffmann, K. et al. Spotting priming-active compounds using parsley cell cultures in microtiter plates. *BMC Plant Biol.* **23**, 72 (2023). 36726070 10.1186/s12870-023-04043-y PMC9893529

[46] Schilling, J. V. et al. Oxygen transfer rate identifies priming compounds in parsley cells. *BMC Plant Biol.* **15**, 282 (2015). 26608728 10.1186/s12870-015-0666-3 PMC4660667

[47] Katz, V., Fuchs, A. & Conrath, U. Pretreatment with salicylic acid primes parsley cells for enhanced ion transport following elicitation. *FEBS Lett.* **520**, 53–57 (2002). 12044869 10.1016/s0014-5793(02)02759-x

[48] Sistenich, A. J., Fürtauer, L., Scheele, F. & Conrath, U. Marker and readout genes for defense priming in *Pseudomonas cannabina* pv. alisalensis interaction aid understanding systemic immunity in *Arabidopsis*. *Sci. Rep.* **14**, 3489 (2024). 38347062 10.1038/s41598-024-53982-5 PMC10861594

[49] Chen, Y.-L. et al. XCP1 cleaves pathogenesis-related protein 1 into CAPE9 for systemic immunity in *Arabidopsis*. *Nat. Commun.* **14**, 4697 (2023). 37542077 10.1038/s41467-023-40406-7 PMC10403534

[50] Wang, Y. et al. Coordinated regulation of plant defense and autoimmunity by paired trihelix transcription factors ASR3/AITF1 in *Arabidopsis*. *New Phytol.* **237**, 914–929 (2023). 36266950 10.1111/nph.18562

[51] Javed, T. & Gao, S. J. WRKY transcription factors in plant defense. *Trends Genet.* **39**, 787–801 (2023). 37633768 10.1016/j.tig.2023.07.001

[52] Katagiri, F., Thilmony, R. & He, S. Y. The *Arabidopsis thaliana*-*Pseudomonas syringae* interaction. *Arab. Book Am. Soc. Plant Biol.* **1**, e0039 (2002). 10.1199/tab.0039 PMC3243347 22303207

[53] Maleck, K. et al. The transcriptome of *Arabidopsis thaliana* during systemic acquired resistance. *Nat. Genet.* **26**, 403–410 (2000). 11101835 10.1038/82521

[54] Herman, M. A., Davidson, J. K. & Smart, C. D. Induction of plant defense gene expression by plant activators and *Pseudomonas syringae* pv. tomato in greenhouse-grown tomatoes. *Phytopathology* **98**, 1226–1232 (2008). 18943412 10.1094/PHYTO-98-11-1226

[55] Cooper, A. & Ton, J. Immune priming in plants: from the onset to transgenerational maintenance. *Essays Biochem.* **66**, 635–646 (2022). 35822618 10.1042/EBC20210082 PMC9528079

[56] Pastor, V., Luna, E., Mauch-Mani, B., Ton, J. & Flors, V. Primed plants do not forget. *Environ. Exp. Bot.* **94**, 46–56 (2013).

[57] Catoni, M. et al. Long-lasting defence priming by β-aminobutyric acid in tomato is marked by genome-wide changes in DNA methylation. *Front. Plant Sci.* **13**, 836326 (2022). 35498717 10.3389/fpls.2022.836326 PMC9051511

[58] Worrall, D. et al. Treating seeds with activators of plant defence generates long-lasting priming of resistance to pests and pathogens. *New Phytol.* **193**, 770–778 (2012). 22142268 10.1111/j.1469-8137.2011.03987.x

[59] Yu, Y. et al. Nematode signaling molecules are extensively metabolized by animals, plants, and microorganisms. *ACS Chem. Biol.* **16**, 1050–1058 (2021). 34019369 10.1021/acschembio.1c00217 PMC8590397

[60] Felix, G., Duran, J. D., Volko, S. & Boller, T. Plants have a sensitive perception system for the most conserved domain of bacterial flagellin. * Plant J.* **18**, 265–276 (1999). 10377992 10.1046/j.1365-313x.1999.00265.x

[61] Chinchilla, D., Bauer, Z., Regenass, M., Boller, T. & Felix, G. The *Arabidopsis* receptor kinase FLS2 binds flg22 and determines the specificity of flagellin perception. * Plant Cell* **18**, 465–476 (2006). 16377758 10.1105/tpc.105.036574 PMC1356552

[62] Zipfel, C. Plant pattern-recognition receptors. *Trends Immunol.* **35**, 345–351 (2014). 24946686 10.1016/j.it.2014.05.004

[63] Mendy, B. et al. Arabidopsis leucine-rich repeat receptor-like kinase NILR1 is required for induction of innate immunity to parasitic nematodes. *PLoS Pathog.* **13**, e1006284 (2017). 28406987 10.1371/journal.ppat.1006284 PMC5391088

[64] Zhi, P. & Chang, C. Exploiting epigenetic variations for crop disease resistance improvement. *Front. Plant Sci.* **12**, 692328 (2021). 34149790 10.3389/fpls.2021.692328 PMC8212930

[65] Giresi, P. G., Kim, J., McDaniell, R. M., Iyer, V. R. & Lieb, J. D. FAIRE (formaldehyde-assisted isolation of regulatory elements) isolates active regulatory elements from human chromatin. *Genome Res.* **17**, 877–885 (2007). 17179217 10.1101/gr.5533506 PMC1891346

[66] Simon, J. M., Giresi, P. G., Davis, I. J. & Lieb, J. D. Using formaldehyde-assisted isolation of regulatory elements (FAIRE) to isolate active regulatory DNA. *Nat. Protoc.* **7**, 256–267 (2012). 22262007 10.1038/nprot.2011.444 PMC3784247

[67] Kumar, B. et al. Maydis leaf blight of maize: update on status, sustainable management and genetic architecture of its resistance. *Physiol. Mol. Plant Pathol.* **121**, 101889 (2022).

[68] Hao, Q. et al. An pair of an atypical NLR encoding genes confer Asian soybean rust resistance in soybean. *Nat. Commun.* **15**, 3310 (2024). 38632249 10.1038/s41467-024-47611-y PMC11023949

[69] Gupta, Y. K. et al. Major proliferation of transposable elements shaped the genome of the soybean rust pathogen *Phakopsora pachyrhizi*. *Nat. Commun.* **14**, 1835 (2023). 37005409 10.1038/s41467-023-37551-4 PMC10067951

[70] Kaliszewicz, A. & Uchmański, J. A cross-phyla response to *Daphnia* chemical alarm substances by an aquatic oligochaete. *Ecol. Res.* **24**, 461–466 (2009).

[71] Pare, P. W., Alborn, H. T. & Tumlinson, J. H. Concerted biosynthesis of an insect elicitor of plant volatiles. *Proc. Natl. Acad. Sci. USA* **95**, 13971–13975 (1998). 9811910 10.1073/pnas.95.23.13971 PMC24993

[72] Newman, M. A., Sundelin, T., Nielsen, J. T. & Erbs, G. MAMP (microbe-associated molecular pattern) triggered immunity in plants. *Front. Plant Sci.* **4**, 139 (2013). 23720666 10.3389/fpls.2013.00139 PMC3655273

[73] Walters, D. R. Are plants in the field already induced? Implications for practical disease control. *Crop Prot.* **28**, 459–465 (2009).

[74] Kunwar, S. et al. Foliar applications of acibenzolar-S-methyl negatively affect the yield of grafted tomatoes in fields infested with *Ralstonia solanacearum*. *Plant Dis.* **101**, 890–894 (2017). 30682942 10.1094/PDIS-03-16-0331-RE

[75] Henikoff, S. Nucleosome destabilization in the epigenetic regulation of gene expression. *Nat. Rev. Genet.* **9**, 15–26 (2008). 18059368 10.1038/nrg2206

[76] Luna, E., Bruce, T. J. A., Roberts, M. R., Flors, V. & Ton, J. Next-generation systemic acquired resistance. *Plant Physiol.* **158**, 844–853 (2012). 22147520 10.1104/pp.111.187468 PMC3271772

[77] Berardini, T. Z. et al. The *Arabidopsis* information resource: making and mining the “gold standard” annotated reference plant genome. *Genesis* **53**, 474–485 (2015). 26201819 10.1002/dvg.22877 PMC4545719

[78] Huang, L. et al. A receptor for dual ligands governs plant immunity and hormone response and is targeted by a nematode effector. *Proc. Natl. Acad. Sci. USA* **121**, e2412016121 (2024). 39388275 10.1073/pnas.2412016121 PMC11494329

[79] Schenk, S. T. et al. N-acyl-homoserine lactone primes plants for cell wall reinforcement and induces resistance to bacterial pathogens via the salicylic acid/oxylipin pathway. *Plant Cell* **26**, 2708–2723 (2014). 24963057 10.1105/tpc.114.126763 PMC4114961

[80] Waldo, B. D., Grabau, Z. J., Mengistu, T. M. & Crow, W. T. Nematicide effects on non-target nematodes in bermudagrass. *J. Nematol.* **51**, 1–12 (2019). 31088021 10.21307/jofnem-2019-009 PMC6929642

[81] Carpio, M. J., García-Delgado, C., Marín-Benito, J. M., Sánchez-Martín, M. J. & Rodríguez-Cruz, M. S. Soil microbial community changes in a field treatment with chlorotoluron, flufenacet and diflufenican and two organic amendments. *Agronomy* **10**, 1166 (2020).

[82] Al-Ani, M. A. M., Hmoshi, R. M., Kanaan, I. A. & Thanoon, A. A. Effect of pesticides on soil microorganisms. *J. Phys. Conf. Ser.* **1294**, 072007 (2019).

[83] Yu, Z., Lu, T. & Qian, H. Pesticide interference and additional effects on plant microbiomes. * Sci. Total Environ.* **888**, 164149 (2023). 37196943 10.1016/j.scitotenv.2023.164149

[84] Jeyaseelan, A., Murugesan, K., Thayanithi, S. & Palanisamy, S. B. A review of the impact of herbicides and insecticides on the microbial communities. *Environ. Res.* **245**, 118020 (2024). 38151149 10.1016/j.envres.2023.118020

[85] Curtis, B. J. et al. Identification of uric acid gluconucleoside-ascaroside conjugates in *Caenorhabditis elegans* by combining synthesis and MicroED. *Org. Lett*. **22**, 6724–6728 (2020). 10.1021/acs.orglett.0c02038 PMC7526323 32820938

[86] Nürnberger, T. et al. High affinity binding of a fungal oligopeptide elicitor to parsley plasma membranes triggers multiple defense responses. *Cell* **78**, 449–460 (1994). 8062387 10.1016/0092-8674(94)90423-5

[87] King, E. O., Ward, M. K. & Raney, D. E. Two simple media for the demonstration of pyocyanin and fluorescin. *J. Lab Clin. Med.* **44**, 301–307 (1954). 13184240

[88] Chomczynski, P. & Sacchi, N. Single-step method of RNA isolation by acid guanidinium thiocyanate-phenol-chloroform extraction. *Anal. Biochem.* **162**, 156–159 (1987). 2440339 10.1006/abio.1987.9999

[89] Livak, K. J. & Schmittgen, T. D. Analysis of relative gene expression data using real-time quantitative PCR and the 2^−ΔΔCT^ method. *Methods* **25**, 402–408 (2001). 11846609 10.1006/meth.2001.1262

[90] Tian, M. et al. *Arabidopsis* actin-depolymerizing factor AtADF4 mediates defense signal transduction triggered by the *Pseudomonas syringae* effector AvrPphB. *Plant Physiol.* **150**, 815–824 (2009). 19346440 10.1104/pp.109.137604 PMC2689984

[91] Manosalva, P. M. et al. Methyl esterase 1 (StMES1) is required for systemic acquired resistance in potato. *Mol. Plant Microbe Interact.* **23**, 1151–1163 (2010). 20687805 10.1094/MPMI-23-9-1151

[92] Calvert, L. & Ghabrial, S. Enhancement by soybean mosaic virus of bean pod mottle virus titer in doubly infected soybean. *Phytopathology* **73**, 992–997 (1983).

[93] Jeffers, S. *Laboratory Protocols for Phytophthora Species Protocols* (ed. Jeffers, S.) 1–2 (The American Phytopathological Society, 2015).

[94] Kachroo, A. et al. An oleic acid-mediated pathway induces constitutive defense signaling and enhanced resistance to multiple pathogens in soybean. *Mol. Plant Microbe Interact.* **21**, 564–575 (2008). 18393616 10.1094/MPMI-21-5-0564

[95] Kauffman, H. E., Reddy, A. P. K., Hsieh, S. P. Y. & Merca, S. D. An improved technique for evaluating resistance of rice varieties to *Xanthomonas oryzae*. *Plant Dis. Rep.* **57**, 537–541 (1973).

[96] Weller, D. M. et al. *Rhizoctonia* root rot of small grains favored by reduced tillage in the Pacific Northwest. *Plant Dis.* **70**, 70–73 (1986).

[97] Okubara, P. A., Leston, N., Micknass, U., Kogel, K. H. & Imani, J. Rapid quantitative assessment of *Rhizoctonia* resistance in roots of selected wheat and barley genotypes. *Plant Dis.* **100**, 640–644 (2016). 30688595 10.1094/PDIS-05-15-0611-SR

[98] Hristova, K. & Wimley, W. C. Determining the statistical significance of the difference between arbitrary curves: a spreadsheet method. *PLoS ONE* **18**, e0289619 (2023). 37906570 10.1371/journal.pone.0289619 PMC10617697

[99] Bisceglia, N. G., Gravino, M. & Savatin, D. V. Luminol-based assay for detection of immunity elicitor-induced hydrogen peroxide production in *Arabidopsis thaliana* leaves. *Bio Protoc.* **5**, e1685 (2015). 10.21769/BioProtoc.1379 PMC5660616 29085857

