# Supplementary material for: The conserved nematode pheromone ascr#18 primes plant immunity
Source: Commun Biol. 2026 May 6;9:936. doi: 10.1038/s42003-026-10211-1 (PMC13350848; doi:10.1038/s42003-026-10211-1)
Supplement: Supplementary file 2 — Supplementary Information [file 42003_2026_10211_MOESM2_ESM.pdf]

SUPPLEMENTARY INFORMATION

The conserved nematode pheromone ascr#18 primes plant immunity

Murli Manohar[1,*], Andrea Sistenich[2,*], Shoashuai Liu[3], Shine Baby[4], Shiyan Chen[5], Wim Dejonghe[6], Anshu Kumari[1], Emily Luna[7], Sophie Levecque[2], Patricia M. Manosalva[8], Jan Leach[7], Xiaohong Wang[9], Aardra Kachroo[4], Karl-Heinz Kogel[3,§], Uwe Conrath[2], Frank C. Schroeder[1], and Daniel F. Klessig[1]

[1]Boyce Thompson Institute, Ithaca, NY 14853, USA.

[2]Department of Molecular Plant Physiology, RWTH Aachen University, Aachen 52056, Germany.

[3]Research Center for BioSystems, Land Use, and Nutrition, Justus Liebig University, Giessen, Germany.

[4]Department of Plant Pathology, University of Kentucky, Lexington, KY 40503, USA.

[5]School of Integrative Plant Science, Cornell University, Ithaca, NY 14853, USA.

[6]Ascribe Bioscience, Ithaca, NY 14850, USA.

[7]Department of Agricultural Biology, Colorado State University, Fort Collins, CO 80523, USA.

[8]Department of Plant Pathology and Microbiology, University of California, Riverside, CA 92507, USA.

[9]Robert W. Holley Center for Agriculture and Health, US Department of Agriculture, Agricultural Research Service, Ithaca, NY 14853, USA.

[§]Present address: Institute of Plant Molecular Biology (CNRS), University of Strasbourg, 12 Rue du Général Zimmer, 67084, Strasbourg, France.

*These authors contributed equally.

Correspondence and requests for material should be addressed to M.M. (mm829@cornell.edu), U.C. (uwe.conrath@bio3.rwth-aachen.de), F.C.S. (schroeder@cornell.edu) or D.F.K. (dfk8@cornell.edu)

Supplementary Figures

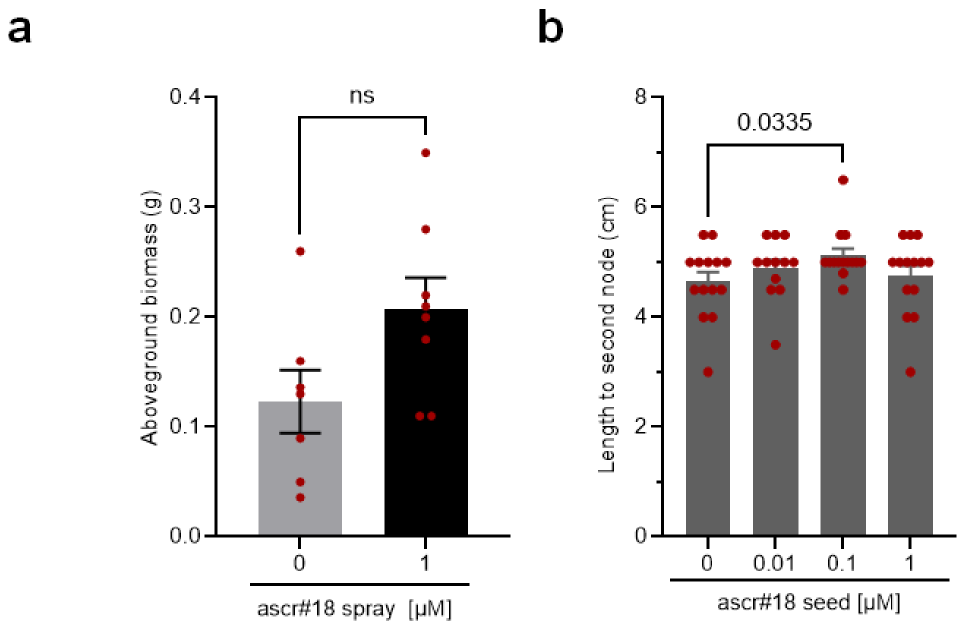

Supplementary Figure S1. Ascr#18 treatment does not reduce growth of *Arabidopsis* and wheat plants. **a,** Twelve-day-old *Arabidopsis* seedlings were spray treated with 1 μM of ascr#18 or 0.1% ethanol (0) solution growing on a 0.5X MS agar plate for 48 h. Seedlings were then transferred to the soil. Measurement of aboveground biomass was taken 30 days post-treatment. Data are averages ±SEM (n ≥7). **b,** Wheat seeds were pretreated for 24 h without (0) or with three concentrations of ascr#18 (0.01, 0.1, and 1 μM) before planting in soil. Plant height was measured 10 days post-planting**.** Data are averages ±SEM (n ≥12). Adjusted *p*-values were calculated using two-tailed *t*-tests.

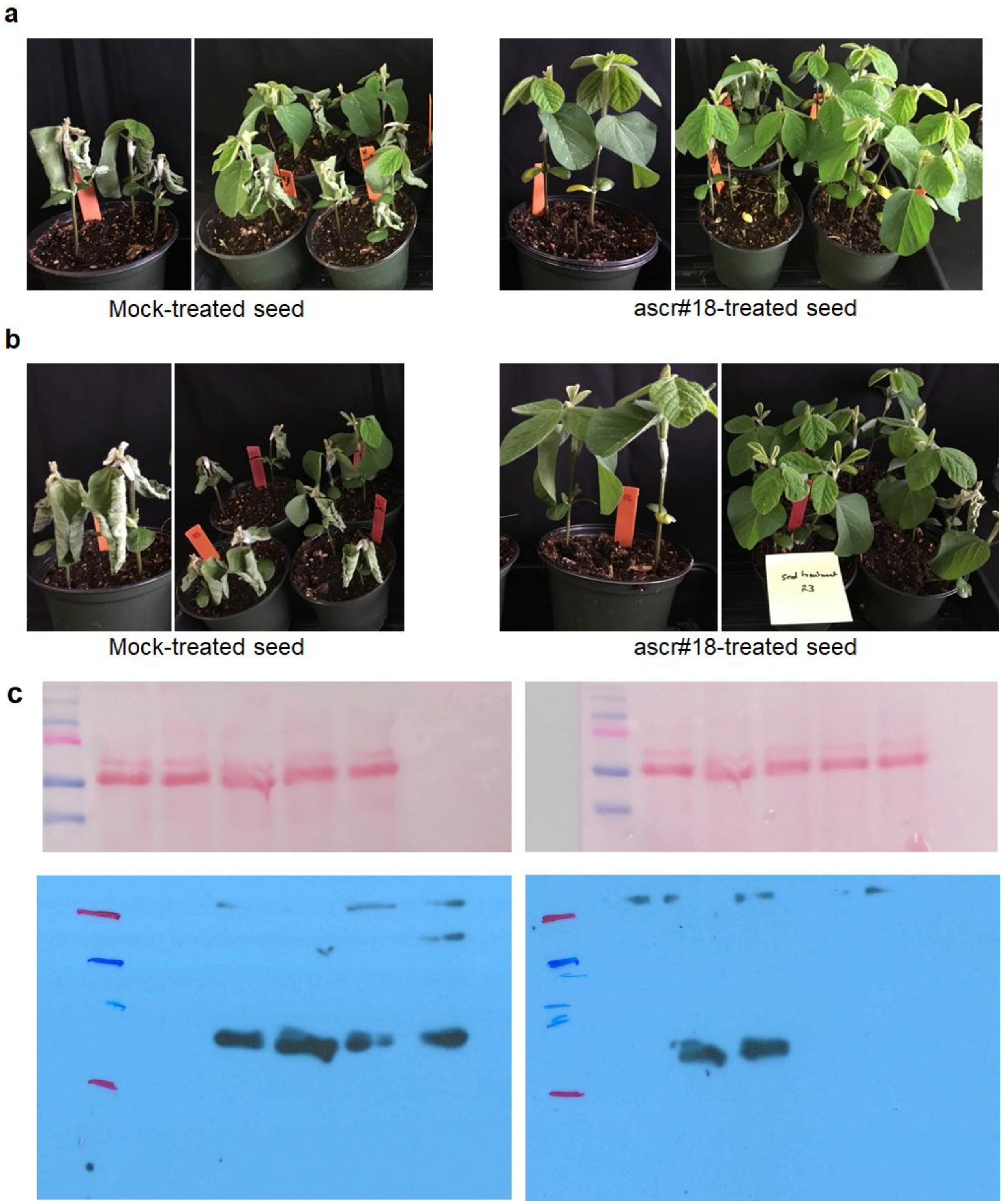

Supplementary Figure S2. Ascr#18-mediated defense priming enhances resistance to two races of *P. sojae*. Soybean plants grown from 100 µM ascr#18- or mock-treated seeds were inoculated with *P. sojae* race 1 (**a**) and race 3 (**b**) at the V1 stage. Representative images comparing disease severity were taken 10 days after inoculation. Plants were grown in 4-inch diameter pots. **c,** Unedited versions of blot images shown in Figure 2f, for mock-treated seed (left) and ascr#18-treated seed (right).

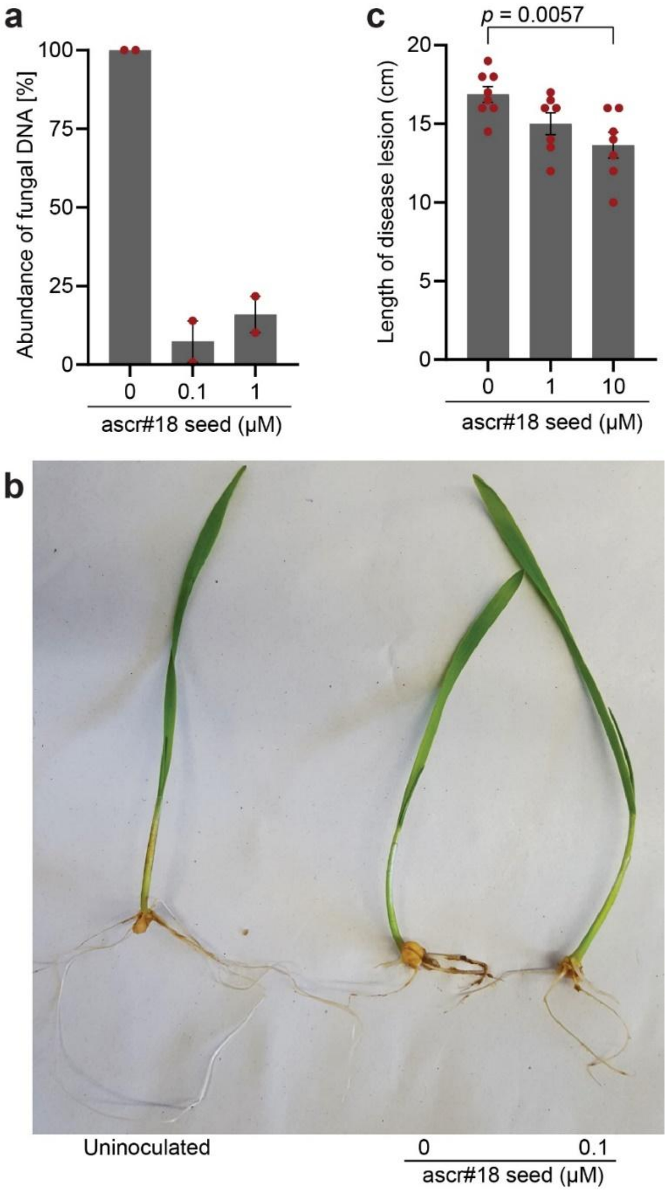

Supplementary Figure S3. Ascr#18-mediated defense priming enhances resistance to fungal and bacterial pathogens. a, Wheat seeds were treated with 0.1 or 1 µM of ascr#18 for 24 h before planting. Two-day-old seedlings were inoculated with the fungus *Rhizoctonia solani.* Abundance of fungal DNA at 12 dpi was measured by quantifying *internal transcribed spacers 1* (*ITS1*) using DNA extracted from infected root tissue. Each treatment included plants grown from 20 treated seeds. Data from three independent experiments were averaged. **b,** Representative image of wheat seedlings 4 dpi showing uninoculated control (left) and infected plants grown from untreated seed (center) or from seeds treated with 0.1 µM ascr#18 (right). **c,** Ascr#18 treatment suppressed bacterial blight on rice caused by *Xanthomonas oryzae* pv. *oryzae*. Three-week-old rice plants (cv. Kitaake) grown from seeds treated with the indicated concentrations of ascr#18 were inoculated with *X. oryzae* pv. *oryzae*. Lengths of lesions were measured 14 dpi to assess disease severity. Data are mean ± SEM ($n \geq 7$). Adjusted *p*-values were calculated using one-way ANOVA. dpi, days post-inoculation.

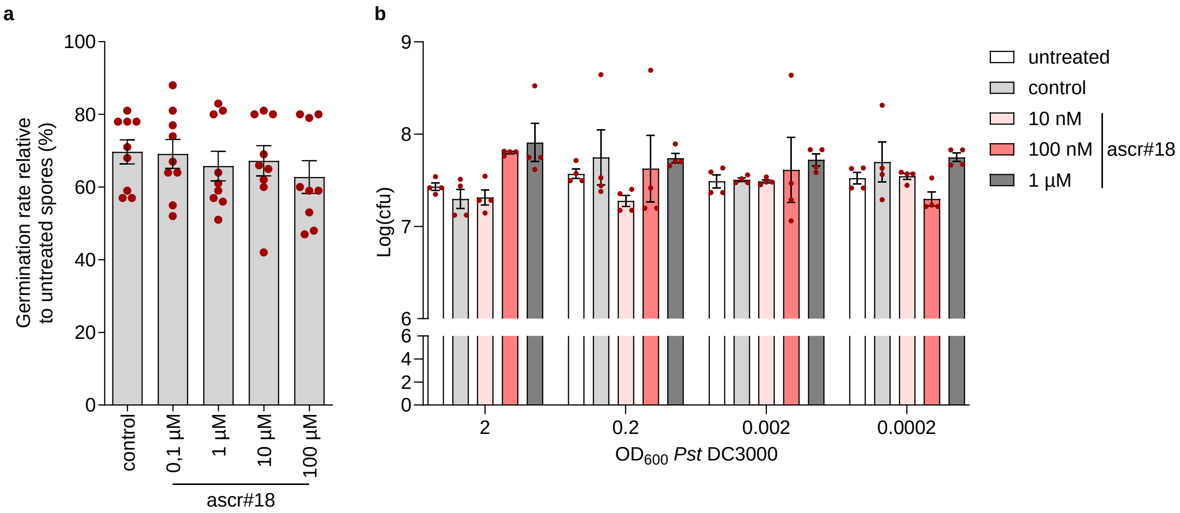

Supplementary Figure S4. Ascr#18 treatment does not directly affect the germination of *P. pachyrhizi* or the multiplication of *Pst* DC3000. a, Aliquots of *P. pachyrhizi* suspension (1 mg/ml) in 0.01% Tween-20 were treated with ethanol (control), ascr#18 (0.1, 1, 10 or 100 µM) in ethanol. After incubation for 3 h at 20 °C, the germination rate of approximately 3 x 100 spores per aliquot was determined relative to untreated spores. Data are mean ± SEM (*n* =9), from 3 independent experiments. **b,** OD_{600} of overnight cultures of ***Pst* DC3000** grown at 28 °C was determined and then diluted to OD_{600} 2, 0.2, 0.002, and 0.0002. 5 ml of each dilution was treated with ethanol (control) or ethanol solutions of ascr#18, resulting in final ascr#18 concentrations of 10, 100 nM, or 1 µM in the media. One aliquot was left untreated. Cultures were incubated overnight at 28 °C and 220 rpm on a rotary shaker before serial dilution and plating of 5 µl drops on Petri dishes containing King's B medium supplemented with 100 µg/ml rifampicin, 25 µg/ml kanamycin, and 10 g/l agar. CFU were determined after 48 h of incubation at 28°C. Data are mean ± SEM (*n* =4), from 4 independent experiments.

Supplementary Tables

Table S1. Changes of *NILR1* expression in response to priming and pathogen challenge (*Pseudomonas syringae* pv. *maculicola* (*Psm*) infection).

ncP, effect of priming in the absence of challenge; npC, effect of challenge in the absence of priming, pC, effect of systemic challenge on priming; cP, effect of priming on systemic challenge., logFC, log fold change; FDR, false discovery rate. Based on a previously published dataset[1].

	**ncP logFC**	ncP FDR	**npC logFC**	npC FDR	**pC logFC**	pC FDR	**cP logFC**	cP FDR	**FAIRE logRatio**	FAIRE p-value
NILR1	2,77	1,41E-06	4,13	4,85E-14	2,91	1,86E-08	1,55	6,27E-03	3,53	6,38E-03

Table S2. Primers for RT-qPCR to measure FAIRE-DNA and input DNA. Position refers to the middle position of the PCR product within the gene starting with start codon (ATG) +1.

Gene	Position	5'-3' sequence	Primer direction
ACTIN2	1032	GGTAACATTGTGCTCAGTGGTGG	forward
		GGTGCAACGACCTTAATCTTCAT	reverse
FRK1	-830	GTATGATCATACATTAATATCAGAATAGTC	forward
		CTTAGTTTCATTGCCATCCAC	reverse
	-715	CATTGACTTAGAAGTCGACAAAAAAAAATA	forward
		GATGATCCGCTTTCAACGATACA	reverse
	-541	GTCACCTACCAATGTGGTTTTGC	forward
		GGAATCAGTTGAATATCAATGTGTTACG	reverse
	-439	CTATTGGGAGTTGTGAGATTTTTTATATC	forward
		CTATATATGCTATAACACCACATCACAAAT	reverse
	-344	CAAAAGGAAATTAGATGTGTTTTGACC	forward
		ACAACGTTGCCAAAAAAAAAAAACTAG	reverse
	200	AAGGAAGCGGTCAGATTTCA	forward
		TTCATTTGGCGAGTCGAATA	reverse
PR1	-650	AAGTCCTGAAGAATATATGCCGCC	forward
		TTTCTTTTTGGATAAATCTCAATGGG	reverse
	-436	TTCAAAATGTTTTGAAGATATCTTCCTG	forward
		TTGCCAAACTGTCCGATACG	reverse
	-173	CCAAATGAATAGAAGTTGTTTTCGTAAC	forward
		GAAAAATGTGTGTAAGGACAAGATTGAC	reverse
	-103	CTATAGATCTCACGTTTTTGTAAATACATG	forward
		TTGCAATGAATATTACTAAAATACTCACAC	reverse
	-35	CTTATATAGAGATTTGAAAACTATTTTTTTC	forward
		ATTTACAAAAACGTGAGATCTATAGTTAAC	reverse
	125	TGCCTGGTTGTGAACCCTTAG	forward
		TCGAAAGCTCAAGATAGCCCAC	reverse
WRKY6	-574	GTTCTACATTATTCTTTTGGCGACG	forward
		AAGTTGTTTACTAGTAATCCGATTGGC	reverse
	-400	TTGGGATTTATGATTAGTTTTTAGGG	forward
		ACCTGAATCATACACTGTAATAAAAAAAAC	reverse
	-145	ATTCTTGGGAGTTTTACGTCAGATC	forward
		GGTCGTAACCGGGCAATG	reverse
	-25	GAAGTCTTCTGCCAGAACATAAGAC	forward
		CGTCCCAAACCCTTTTTATAGG	reverse
	334	ACTTCACGGTCATTATCTCCAGC	forward
		TGAATTTAGGTTTCCGGTGAGTC	reverse

WRKY29	-850	TGGGATTAAAGGTATCCATCCCTAT	forward
		TCACTTCATCTTAAGCCTTGTGGT	reverse
	-644	GTATAATTATGTATGTGTTTAATGGGGCAC	forward
		GTTACGTGAACACAAGGTAAGCTAATAGAC	reverse
	-544	CGATCAAATAAAGAAATTACCATGACG	forward
		ATGCATATGCATGAGTATGACATATAGTAG	reverse
	-307	TGGACTTTCCTGAATGTTGTGTATG	forward
		CATTAACTTGTCAAATATCCATCTCCG	reverse
	-170	AAATTTTAAAAGTTTAATGACTTAACGGG	forward
		GATTAGATTGTTTGTGGTTTAGGGTG	reverse
	100	TTTCACCTTCGTTTTGCCTACC	forward
		CGAGCTCATCTAAGCCACTTGTC	reverse
WRKY53	-392	GAATATTTTCAAAATATGTTGGTTGATG	forward
		GCAATGAAATATTATCCATAGAAAGGTTG	reverse
	-250	CAACAAAGGGTTATACAACTGTCTCTTTC	forward
		AGAGAATCTTAATCACATGTACAAAGTCG	reverse
	-100	GGTATAAGGAAGTATAGGGCCAGAGTG	forward
		CAAAATTGAAATGTTGAATTGGTCAG	reverse
	70	TAGGATTGGTGATAAAAATGGATGAG	forward
		TTTTCAAACACTGAAAATCCAATGC	reverse
	417	GCTAAGCGAGCTTATCAATGG	forward
		GACGGAGCTTCTCTAAGTC	reverse

Table S3. Primers for RT-qPCR to measure mRNA transcript abundance.

Gene	Locus	5’-3’ sequence	Primer direction
ACTIN2	At3g18780	GGTAACATTGTGCTCAGTGGTGG	forward
		GGTGCAACGACCTTAATCTTCAT	reverse
β-tubulin	At5g44340	GTCCAGTGTCTGTGATATTGCACC	Forward
		TTACGAATCCGAGGGAGCCATTG	Reverse
FRK1	At2g19190	AAGGAAGCGGTCAGATTTCA	forward
		TTCATTTGGCGAGTCGAATA	reverse
PR1	At2g14610	GGTAGCGGTGACTTGTCTGG	forward
		CAAACTCCATTGCACGTGTT	reverse
WRKY6	At1g62300	ACTTCACGGTCATTATCTCCAGC	forward
		TGAATTTAGGTTTCCGGTGAGTC	reverse
WRKY29	At4g23550	TTTCACCTTCGTTTTGCCTACC	forward
		CGAGCTCATCTAAGCCACTTGTC	reverse
WRKY53	At4g23810	CTCCATCGGCAAACTCTTCAC	forward
		CCGAGCGTACAACTTATTCCG	reverse

Supplementary Reference

1 Baum, S. *et al.* Isolation of Open Chromatin Identifies Regulators of Systemic Acquired Resistance. *Plant Physiol* **181**, 817-833, doi:10.1104/pp.19.00673 (2019).
