# Supplementary material for: The conserved nematode pheromone ascr#18 primes plant immunity
Source: Commun Biol. 2026 May 6;9:936. doi: 10.1038/s42003-026-10211-1 (PMC13350848; doi:10.1038/s42003-026-10211-1)
Supplement: Supplementary file 3 — Description of Additional Supplementary Files [file 42003_2026_10211_MOESM3_ESM.pdf]

Description of Additional Supplementary Files

File name: Supplementary Data 1

Description: The source data behind the graphs in the paper
