# Supplementary material for: The conserved nematode pheromone ascr#18 primes plant immunity
Source: Commun Biol. 2026 May 6;9:936. doi: 10.1038/s42003-026-10211-1 (PMC13350848; doi:10.1038/s42003-026-10211-1)
Supplement: Supplementary file 1 — Transparent Peer Review File [file 42003_2026_10211_MOESM1_ESM.pdf]

nature portfolio

Peer Review File

The conserved nematode pheromone ascr#18 primes plant immunity

Corresponding Author: Professor Frank Schroeder

This file contains all reviewer reports in order by version, followed by all author rebuttals in order by version.

Version 0:

Reviewer comments:

Reviewer #1

(Remarks to the Author)
The authors describe nematode pheromone ascr#18-triggered defense priming of plants, particularly Arabidopsis, against a variety of pathogens. They claim that Ascr#18 does not initiate pattern-triggered immunity (PTI) response alone but will trigger PTI when a plant is inoculated with pathogen. The priming effect is seen in both greenhouse and field conditions, and crop health is significantly improved upon ascr#18 treatment. The authors indicate that this priming occurs by ascr#18-mediated opening of chromatin in the promoter region of typical defense response genes, and that the cell surface receptor of ascr#18, NILR1, is required for this. The manuscript is well-written. Their findings provide intriguing insights into plant-nematode interactions and the value of this research is well-demonstrated through field application. I have a few concerns/questions that need to be addressed prior to publication.
Major comments:
1. Figure 1 C-F: The timing of this experiment is a bit unclear. Is the total time from ascr#18 treatment 24h or 30h? Please clarify this in the figure legend. Additionally, multiple previously published papers have demonstrated significant defense gene induction upon ascr#18 treatment alone (Huang, 2023; Manohar, 2020; Manosalva, 2015), but the information presented here contradicts this. Please offer an explanation to these discrepancies, perhaps in the Discussion section of this manuscript. Finally, how are the data normalized in this figure, and would an adjustment to this normalization correspond with findings from published literature (e.g. Control no Pst inoculation versus ACTIN2 alone)?
2. Figure 3A: At the time point tested, ascr#18 alone appears to induce immune gene expression (at least by just looking at the bars, particularly for WRKY29 & WRKY6), though further inoculation of P. syringae significantly enhanced such activity. Please clarify the discrepancy between Figures 1 C-F and Figure 3 A. Is there a difference due to the normalization/analysis used in these different figures (relative fold change vs. relative transcript abundance; ACTIN2 vs. B-tubulin)?
3. Based on previous publications from the authors' and other labs and data shown in Figure 3A. Ascr#18 certainly can directly induce defense responses (or PTI), I don't think the authors can rule out the possibility that ascr#18-primed defense works in parallel with or in addition to Ascr18-NILR1 PTI to defend against invading pathogens. I would like the authors to try to investigate other mechanisms underlying ascr#18 priming, particularly induction of PRR genes (e.g. FLS2) and MAPK3/6 as they stated in the Introduction – or at least discuss these possibilities in the Discussion.
4. Figure 4e: Is NILR1 required for ascr#18-mediated chromatin modification as shown in the model? – I assume so! To definitively demonstrate this, it would be necessary to conduct FAIRE qPCR assay using one of the nilr1 mutants as they did in Figure 3b.

Minor points:
1. In the section "Ascr#18 primes Arabidopsis plants for enhanced defense gene expression" describing Figure 1, citations 32 and 33 (Manosalva 2015 and Manohar 2020) are incorrectly referenced, as both publications found induced defense gene expression (FRK1, PR1, WRKY53, and others) at the experimental timepoint. Please reword this accordingly.
2. Figure 4: What is the measure of disease severity? Please include this in the figure legend and/or clarify what "visually" means in the "Field Testing" part of the Methods section.
3. Please clarify what "n=" specifically refers to in each of the figures: biological or technical replicates.
4. Page 10, the third paragraph (… the recently identified ascr#18 receptor NILR1, a dedicated LRR-RLK that has orthologues in a wide range of dicotyledonous as well as monocotyledonous species.). It has been recently published that Ascr#18 is also perceived by the potato NILR1 ortholog. It would be helpful to cite the publication: A receptor for dual ligands governs plant immunity and hormone response and is targeted by a nematode effector. Huang et al., Proc Natl Acad Sci U S A. 2024 Oct 15;121(42):e2412016121. doi: 10.1073/pnas.2412016121. Epub 2024 Oct 10.

Reviewer #2

(Remarks to the Author)
Review of COMMSBIO-24-8208-T

This paper by Manohar et al. Describes the use of Ascr#18 to prime plants for enhanced defence against pathogens. This paper is a follow-up of previous papers from the same research group showing that this nematode pheromone activates PTI in various plant species, and can protect them from a diversity of pathogens and pests.

Major comments:
- The fact that already at LEAST 3 previous papers from the group have shown the immune-stimulating activity of this ascaroside is a major issue. This manuscript fails to provide the reader with novel insights. What are the additional research questions that were answered in the current manuscript? What new information is provided here?
- The experimental procedures used in this manuscript vary too much. The applied Ascr#18 concentration varies with a factor of at least 100 between experiments, see for example figure 2b vs 2c (and many others listed below). These differences are not justified or discussed by the authors.

Minor comments
- Page 3: the second paragraph says that MAMPs can prime plant immune responses. Not only MAMPs can do that. What about chemical priming agents, such as BABA, etc.?
- It is not clear to me why SA was chosen here as positive control for priming. SA is a known plant hormone, that will activate all SA responsive genes directly. PR genes for example are known to directly respond to SA treatment. Indeed, on page 9 the authors claim themselves that acibenzolar-S-methyl (SA-analogue) directly induces plant defence genes.
- Why is 30 nM less effective in the plant cells than 15 nM? Is the high concentration phytotoxic?
- Page 4: the authors suggest that ascr#18 may prime plant defence responses via conserved mechanisms. What is the evidence for this? What is meant by ‘conserved mechanisms’?
- Which ascr#18 concentration was used in the Arabidopsis root drench experiments? Why was this concentration chosen?
- 1 µM Ascr#18 Treated Arabidopsis seeds were inoculated at 5 weeks old. Why this concentration? And moreover, why this time-point? To add novelty to the paper, a time-course experiment would have be instrumental.
- The pathogens, crops and Ascr#18 concentrations used in this manuscript seem to be randomly chosen. It is not well-justified why they were selected.
- On page 7, the authors seem to suggest that histone acetylation is monitored by the FAIRE approach. However, in reality, euchromatin regions are picked-up by this technique. Euchromatin is modified by many more epigenetic modifications than just histone acetylation. This should be better explained.
- In the field experiments, ascr#18 was sprayed on the plants. But all other experiments in this manuscript use seed or root treatment. Why foliar treatment in the field? Was the compound formulated to allow for example uptake in the leaves or rain-fastness? It is again remarkable that the treatments (number and dose) are varying between corn and soybean. Why?
- Page 9: the authors state that their is ‘sustained enhancement of immune gene expression’. This cannot be concluded based on only one time point.
- Page 10: the paragraph starting with ‘our results suggest that ...’ is very confusing and not well-justified by the current dataset.
- It has already been shown that non-plant-parasitic nematodes, such as entomopathogenic nematodes can also activate plant immune response (Shokofeeh Kamali et al, Molecular Ecology 2022). In light of this knowledge, I don’t understand the statement that ‘lack of defence priming by free-living nematodes could lead to increased plant susceptibility’. This statement is not supported by data provided in this manuscript, or in literature.
- How many independent data points were evaluated in the infection experiments?
- How much RNA was used for the qRT-PCRs, in µg?
- Using only one reference gene is a major problem in qRT-PCR. The MIQE guidelines (Bustin et al., 2009), which are nowadays well-accepted and followed by numerous research groups, prescribe to use at least 2 reference genes for accurate quantification.

Reviewer #3

(Remarks to the Author)
This well-written manuscript reports on an impressive amount of work from independent research groups. The paper describes the resistance-inducing activity of the nematode pheromone ascr#18, which was previously found to act as a nematode-associated molecular pattern (NAMP) with PTI-eliciting activity in Arabidopsis and to be perceived by the receptor-like kinase NILR1. The scientific novelty of the current study lies in the demonstration that treatment of taxonomically unrelated plants with relatively low concentrations of this compound induces long-lasting resistance against (hemi)biotrophic pathogens, which in some cases was maintained through the life cycle of plants and was associated with transcriptional priming of salicylic acid (SA)-inducible genes. The work combines high-quality experiments on cell suspension cultures and the genetic model plant Arabidopsis thaliana with field experiments of high translational value. The narrative of the Introduction and Results section is excellent and is supported by a detailed and clearly explained Methods section (barring some details – see my comments below). The Discussion is of equally high value but would perhaps have benefited from articulating the value of the ascr#18-Arabidopsis system as a new model system to study epigenetic plant immune memory.

In summary, I enjoyed reading this inspiring manuscript. Although it would be nice if the authors had experimental data to address some of my questions below, I must stress that these comments should not warrant major revisions. My questions come from a place of curiosity and admiration; in fact, as an editor of a plant journal, I would like to congratulate the editor on having the opportunity to publish a study of such outstanding quality!

Comments:

1. In Arabidopsis, seed treatment with ascr#18 induced long-lasting resistance in fully expanded leaves of 5-week-old plants, which was associated with chromatin relaxation in upstream promoter regions of SA-inducible genes (e.g., PR1, FRL1, WRKYs). I therefore wondered whether the authors have tested mutants in SA induction or perception (e.g., sid2, npr1) against Pseudomonas syringae pv. tomato DC3000 or downy mildew (Hyaloperonospora arabidopsidis, Hpa). This would establish a causal contribution of the transcriptional priming to the induced resistance. Additionally, as reported for other priming agents (e.g. BABA), it is possible that the contribution of SA to the induced resistance depends on the challenging pathogen.

2. The long-lasting nature of ascr#18-induced resistance can best be explained by somatic transmission of an epigenetic priming signal. Indeed, the FAIRE-qPCR results support an epigenetic basis for the long-lasting induced resistance. In this context, I expected a short paragraph in the Discussion that articulates the value of the ascr#18-Arabidopsis system as a model for studying epigenetic immune memory. Additionally, it would be great if the authors included a bit more discussion about the nature of the epigenetic signal driving the somatic transmission of the priming. A brief glance at the integrated genome viewer (JBrowse; TAIR) shows that the five defence genes exhibiting long-term transcriptional priming do not have significant DNA methylation in their promoter regions. If reduced DNA methylation is the epigenetic signal responsible for somatic transmission of the priming (as proposed in previous studies), it would unlikely act via cis-acting mechanisms. Furthermore, I noticed that most of their gene bodies exhibit high levels of H2A.Z, H3K4me, and/or H3K27me3. Could these chromatin modifications play a role?

3. The legend of Figure 3 for the FAIRE-qPCR results mentions “adjusted" p-values. According to the Methods section, these values are either based on ANOVA or two-tailed t-tests. However, it is unclear how the “adjusted” p-values were calculated, as only two conditions (control versus primed) are being compared. Assuming t-tests were performed, how did you account for the different promoter locations? Did you pool values from all promoter locations per treatment? While I have no doubts about the validity of the results, my impression is that simplistic linear models may not be the most appropriate approach to testing for statistical significance in this case. I suspect that more complex nested (generalized) linear models would be more appropriate.

4. In the Methods section, please indicate the source of the ascr#18 compound.

Version 1:

Reviewer comments:

Reviewer #1

(Remarks to the Author)
The authors have addressed my questions, and I am satisfied. I now recommend the manuscript for publication.

Reviewer #3

(Remarks to the Author)
The authors have done a good job addressing my questions and I have no further questions/comments. I therefore strongly support publication of this manuscript without further delays.

Reviewer #4

(Remarks to the Author)
Reviewer #2 (Remarks to the Author): Review of COMMSBIO-24-8208-T

As the newly assigned reviewer, I have re-assessed the manuscript and generally concur with the observations of the previous reviewer. While the authors have made efforts to address the raised questions, the manuscript remains unclear in several areas. In certain instances, the authors appear to contradict their own prior observations, which may contribute to persistent confusion and ambiguity. My assessment is as follows:

- Many of the experiments presented are not novel, as they closely replicate findings from the authors’ previous work. Additionally, the observed potency of ascr#18 in inducing defense genes appears inconsistent. For example, the results shown in Figure 1d differ from earlier publications: in the present study, ascr#18 alone does not significantly induce FRK1 and PR1, whereas previous work identified this as a key finding. The inconsistencies regarding defense gene expression with ascr#18 alone and in combination with Pst infection, along with appropriate controls, warrant a detailed RNA-seq analysis.

- The experiments addressing the priming effect and the associated epigenetic mechanisms do not fully resolve the issue of novelty. The introduction of the concept of promoter opening in chromatin regions appears somewhat abrupt. The authors are encouraged to clarify the rationale for proposing that this epigenetic mechanism may be involved. Are there NGS datasets directly supporting this phenomenon? If not, it would be beneficial to explain in the introduction how chromatin remodeling could be relevant in this context. In lines 64–68, the manuscript describes how defense priming in general may be linked to epigenetic programming. Are there literature sources that demonstrate or propose a critical role for epigenetic remodeling in ascr#18-mediated pathogen defense priming?

-In line 69, the authors state that priming induces PPR accumulation. If this priming mechanism is proposed to be NILR1-dependent, it would be important to provide evidence of significant induction of NILR1 upon ascr#18 treatment, either through RT-qPCR or RNA-Seq (as suggested earlier), particularly given the absence of prior reports on this aspect. Furthermore, as NILR1 has been reported to be promiscuous in recognizing multiple pathogens, the authors should clarify how the requirement of the LRR-RLK NILR1 for ascr#18-mediated priming can be distinguished from shared downstream immune responses that occur across different pathogens following recognition. This point is especially relevant because Figure 3 shows reduced expression of defense genes (PR1, FRK1, and WKRY6) in Pst-treated plants in nilr1 mutants as well.

-The use of a single reference gene (ACTIN2) remains questionable. Citing similar results from other studies using different reference genes is not a sufficient justification. Please repeat the RT-qPCR with at least one additional reference gene and normalize data using the geometric mean of ACTIN2 and the additional gene(s), or provide evidence that ACTIN2 was the only stable reference under all conditions. In Manohar et al., 2020 (Nature Communications), UBQ10 was used. Please explain the rationale for switching to ACTIN2 and include a supplementary table with raw Ct values for ACTIN2 from this study and UBQ10 from the previous one to avoid confusion.

- In their last study, the authors discuss that ascr#18 can be metabolized to ascr#9. Is this mechanism still valid when seeds are primed with it ? Or there is an alternative pathway that is switched on?

Version 2:

Reviewer comments:

Reviewer #5

(Remarks to the Author)
The manuscript titled “A conserved nematode pheromone primes plant immunity” by Manohar et al. addresses a highly relevant topic. By investigating the function of a nematode-secreted pheromone (ascr#18), the authors make an important contribution toward bridging the gap between fundamental and applied sciences.

The manuscript is well written, with high readability and an excellent presentation of the results.

As the newly assigned reviewer, I have reassessed the previously raised concerns and am satisfied with how the authors have addressed the earlier comments. I therefore recommend the manuscript for publication.

Open Access This Peer Review File is licensed under a Creative Commons Attribution 4.0 International License, which permits use, sharing, adaptation, distribution and reproduction in any medium or format, as long as you give appropriate credit to the original author(s) and the source, provide a link to the Creative Commons license, and indicate if changes were made.
In cases where reviewers are anonymous, credit should be given to 'Anonymous Referee' and the source.
The images or other third party material in this Peer Review File are included in the article’s Creative Commons license, unless indicated otherwise in a credit line to the material. If material is not included in the article’s Creative Commons license and your intended use is not permitted by statutory regulation or exceeds the permitted use, you will need to obtain permission directly from the copyright holder.
To view a copy of this license, visit https://creativecommons.org/licenses/by/4.0/

Response to reviewers' comments to COMMSBIO-24-8208-T:
"**A conserved nematode pheromone primes plant immunity**"

We thank all three reviewers for their comments and helpful suggestions.

Reviewer #1 (Remarks to the Author):

The authors describe nematode pheromone ascr#18-triggered defense priming of plants, particularly Arabidopsis, against a variety of pathogens. They claim that Ascr#18 does not initiate pattern-triggered immunity (PTI) response alone but will trigger PTI when a plant is inoculated with pathogen. The priming effect is seen in both greenhouse and field conditions, and crop health is significantly improved upon ascr#18 treatment. The authors indicate that this priming occurs by ascr#18-mediated opening of chromatin in the promoter region of typical defense response genes, and that the cell surface receptor of ascr#18, NILR1, is required for this. The manuscript is well-written. Their findings provide intriguing insights into plant-nematode interactions and the value of this research is well-demonstrated through field application. I have a few concerns/questions that need to be addressed prior to publication.

Major comments:

1. Figure 1 C-F: The timing of this experiment is a bit unclear. Is the total time from ascr#18 treatment 24h or 30h? Please clarify this in the figure legend. Additionally, multiple previously published papers have demonstrated significant defense gene induction upon ascr#18 treatment alone (Huang, 2023; Manohar, 2020; Manosalva, 2015), but the information presented here contradicts this. Please offer an explanation to these discrepancies, perhaps in the Discussion section of this manuscript. Finally, how are the data normalized in this figure, and would an adjustment to this normalization correspond with findings from published literature (e.g. Control no Pst inoculation versus ACTIN2 alone)?

Response: We revised Figures 1c and 1e to clarify this. Plants were treated on their roots with ascr#18 for 24 hours before pathogen (or mock) inoculation. Six hours after inoculation, leaf samples were collected for RNA extraction.

Ascr#18, just like other priming agents such as salicylic acid, only partially induces plant defenses with a subset of genes being activated/induced directly to detectable levels, whereas other immune genes are only activated upon subsequent challenge by a pathogen (Sistenich et al., 2024). We revised the Discussion to directly address this point: ""

"Pretreatment of *Arabidopsis* roots or seeds with ascr#18 enhances the expression of a subset of immune genes upon pathogen challenge. Notably, some immune genes exhibit modest direct induction by ascr#18, followed by a further increase in expression after pathogen challenge. Growth conditions can affect whether these genes exhibit only priming or both direct induction and priming. *WRKY6* and *WRKY29* illustrate this well; in the experiment shown in Figure 1, both genes are only primed, whereas in Figure 3 both are directly induced and primed."

The RT-qPCR data for all measured genes and treatments shown in the figure were normalized to ACTIN2 mRNA transcript levels, using the 2(-Delta Delta C(T)) method (Livak and Schmittgen (2001)), reference 81. Previous studies in the Conrath lab produced similar results regardless of whether normalization was done using ACTIN2 or UBIQUITIN5.

2. Figure 3A: At the time point tested, ascr#18 alone appears to induce immune gene expression (at least by just looking at the bars, particularly for WRKY29 & WRKY6), though further inoculation of P. syringae significantly enhanced such activity. Please clarify the discrepancy between Figures 1 C-F and Figure 3 A. Is there a difference due to the normalization/analysis used in these different figures (relative fold change vs. relative transcript abundance; ACTIN2 vs. B-tubulin)?

Response: Please see our answer above, which we now have also addressed in the Discussion section. Also note that we have not observed significant differences in results were using ACTIN2 vs UBIQUITIN5 for normalization.

3. Based on previous publications from the authors' and other labs and data shown in Figure 3A. Ascr#18 certainly can directly induce defense responses (or PTI), I don't think the authors can rule out the possibility that ascr#18-primed defense works in parallel with or in addition to Ascr18-NILR1 PTI to defend against invading pathogens. I would like the authors to try to investigate other mechanisms underlying ascr#18 priming, particularly induction of PRR genes (e.g. FLS2) and MAPK3/6 as they stated in the Introduction – or at least discuss these possibilities in the Discussion.

Response: As stated above, the level of direct plant defense gene induction by ascr#18 appears to depend on the exact growth conditions. We agree with your assessment that ascr#18 activation and/or priming may occur through multiple pathways, and we have discussed this possibility in our previous manuscript, Manohar et al., 2020 (reference 35), and now mention this possibility in the Discussion section.

4. Figure 4e: Is NILR1 required for ascr#18-mediated chromatin modification as shown in the model? – I assume so! To definitively demonstrate this, it would be necessary to conduct FAIRE qPCR assay using one of the nilr1 mutants as they did in Figure 3b.

Response: We anticipate that this is the case, as both ascr#18-mediated defense priming (Fig. 3a) and disease protection are compromised in the mutant (Huang et al., 2023, reference 36).

We agree with the reviewer that FAIRE-qPCR in the *nilr1* mutant background would provide more mechanistic insight into whether NILR1 is required for ascr#18-mediated chromatin remodeling. Unfortunately, we are currently unable to repeat this experiment, as Andrea Sistenich, the PhD student who developed and conducted the FAIRE-qPCR assays, has left the laboratory, and we do not currently have staff with the required expertise.

To address the reviewer's point as thoroughly as possible under the circumstances, we have added relevant data and discussion to the manuscript (see lines 200-208 in the revised manuscript), showing that *NILR1* itself is a target of pathogen-induced priming. Specifically, we analyzed our previously published data and found that local *Pseudomonas syringae* pv. *maculicola* (Psm) infection leads to chromatin opening in the *NILR1* promoter and upregulation of *NILR1* expression in systemic leaves. Notably, this expression does not further increase upon subsequent challenge, which is consistent with a priming response. These findings support the

conclusion that *NILR1* plays an integral role in immune priming and suggest that it may also participate in broader chromatin-level regulation during immune memory. We discuss this parallel in the context of other MAMP receptors such as *FLS2* and *CERK1*, which have similarly been shown to be primed by salicylic acid signaling (Tateda et al., 2014).

Minor points:

1. In the section “Ascr#18 primes Arabidopsis plants for enhanced defense gene expression” describing Figure 1, citations 32 and 33 (Manosalva 2015 and Manohar 2020) are incorrectly referenced, as both publications found induced defense gene expression (FRK1, PR1, WRKY53, and others) at the experimental time point. Please reword this accordingly.

Response: The reviewer is correct. We revised accordingly.

2. Figure 4: What is the measure of disease severity? Please include this in the figure legend and/or clarify what “visually” means in the “Field Testing” part of the Methods section.
3. Please clarify what “n=” specifically refers to in each of the figures: biological or technical replicates.

Response: Disease severity was ranked on a 0-100% scale, based on the percentage of the diseased leaf area relative to the total leaf area as recorded through visual assessment for leaves from each plant. This is standard procedure used by contract research organizations to assess disease in field plots. "n” represents the number of field plots per treatment. We have revised the methods section to clarify these points.

4. Page 10, the third paragraph (… the recently identified ascr#18 receptor NILR1, a dedicated LRR-RLK that has orthologues in a wide range of dicotyledonous as well as monocotyledonous species.). It has been recently published that Ascr#18 is also perceived by the potato NILR1 ortholog. It would be helpful to cite the publication: A receptor for dual ligands governs plant immunity and hormone response and is targeted by a nematode effector. Huang et al., Proc Natl Acad Sci U S A. 2024 Oct 15;121(42):e2412016121. doi: 10.1073/pnas.2412016121. Epub 2024 Oct 10.

Response: Thank you for the suggestion. We have added this important citation (which appeared after initial submission of the manuscript) .

Reviewer #2 (Remarks to the Author):

Review of COMMSBIO-24-8208-T

This paper by Manohar et al. describes the use of Ascr#18 to prime plants for enhanced defence against pathogens. This paper is a follow-up of previous papers from the same research group showing that this nematode pheromone activates PTI in various plant species, and can protect them from a diversity of pathogens and pests.

Major comments:

- The fact that already at LEAST 3 previous papers from the group have shown the immune-stimulating activity of this ascaroside is a major issue. This manuscript fails to provide the reader with novel insights. What are the additional research questions that were answered in the current manuscript? What new information is provided here?

Response: Thank you for this question. Our previous papers showed immune-stimulating properties of ascr#18 and its role in enhancing plant defenses against a wide variety of pathogens. In the current manuscript, we demonstrate that ascr#18 primes a subset of immune genes for enhanced expression upon pathogen challenge by opening the chromatin in their promoter regions (Fig. 3b-c).

Further, we show that this priming is "long-lived" imparting both enhanced immune gene expression upon pathogen challenge (Fig. 1e,f) and provides enhanced disease protection observed against bacterial, fungal and oomycete pathogens in Arabidopsis, tomato and soybean (Fig. 2b-f). To the best of our knowledge, ascr#18 is the only naturally occurring small molecule for which it has been demonstrated that seed treatment at nanomolar to low micromolar concentrations confers robust and long-lasting defense priming in adult plants. Lastly, this is the first time reporting the translational impact of the research, by showing efficacy under real-world field conditions with both the important dicotyledonous and monocotyledonous crops soybean and corn, respectively (Fig. 4).

- The experimental procedures used in this manuscript vary too much. The applied Ascr#18 concentration varies with a factor of at least 100 between experiments, see for example figure 2b vs 2c (and many others listed below). These differences are not justified or discussed by the authors.

Response: This is an important point. Our research, as detailed in references 32-34, indicates that the optimal concentrations of ascr#18 varies significantly between different crops and depends on the mode of application. For instance, the optimal concentration for spray treatment of Arabidopsis is 1 μM, whereas in solanaceous crops like tomato and potato, it ranges from 1 to 10 nM. Such variability in sensitivity is not unusual; for example responses to flg22 vary significantly between plant species, and even between closely related species, see e.g., here https://pmc.ncbi.nlm.nih.gov/articles/PMC3391249/, and tomato FLS2 is about 10-fold more sensitive to flg22 than Arabidopsis FLS2 (https://pmc.ncbi.nlm.nih.gov/articles/PMC3442597/). Likely, the optimal concentration for each crop varies due to several factors, including leaf morphology, molecular penetration, and the kinetics and/or numbers of receptors involved in sensing ascr#18. We have added a sentence in the Discussion section to address this variability (lines 296-297).

Minor comments

- Page 3: the second paragraph says that MAMPs can prime plant immune responses. Not only MAMPs can do that. What about chemical priming agents, such as BABA, etc.?

Response: We have revised the sentence to include additional molecules that are recognized for their defense priming activity, e.g., BABA.

- It is not clear to me why SA was chosen here as positive control for priming. SA is a known plant hormone, that will activate all SA responsive genes directly. PR genes for example are known to directly respond to SA treatment. Indeed, on page 9 the authors claim themselves that acibenzolar-S-methyl (SA-analogue) directly induces plant defence genes.

Response: Note that SA at high concentrations induces defense gene expression, while at lower concentrations it primes defense gene expression (see, e.g. Thulke and Conrath, Salicylic acid has a dual role in the activation of defense-related genes in parsley; DOI: 10.1046/j.1365-313X.1998.00093.x). SA was selected due to its established effectiveness as a defense gene primer in plants. Furthermore, the parsley system is optimized for SA-mediated defense priming. As illustrated in Figure 1b, a low concentration of SA does not significantly induce furanocoumarin accumulation without treatment with the elicitor. In addition to SA, some SA analogs, such as acibenzolar-S-methyl, are known to similarly prime defense responses (Katz et al., 1998; A benzothiadiazole primes parsley cells for augmented elicitation of defense responses; DOI: 10.1104/pp.117.4.1333).

- Why is 30 nM less effective in the plant cells than 15 nM? Is the high concentration phytotoxic?

Response: As we have shown previously (see e.g., Manosalva et al. 2015; reference 34), responses to ascr#18 often follow a bell-shaped pattern, meaning there is an optimal concentration for effectiveness, while concentrations both above and below this peak are less effective. We have observed this in most of plant host-pathogen systems we and our collaborators have tested over the past two decades. The reason for the observed decrease in activity at higher ascaroside concentrations is not entirely clear. It has been suggested that elevated levels of small-molecule ligands may lead to unproductive receptor engagement. This could occur by disrupting the formation of receptor dimers, which are necessary for ascaroside perception via GPCRs in C. elegans. At none of the concentrations used in our studies did we observe any indication for phytotoxicity.

- Page 4: the authors suggest that ascr#18 may prime plant defence responses via conserved mechanisms. What is the evidence for this? What is meant by 'conserved mechanisms'?

Response: The reviewer is correct; the reference to conserved defense pathways (e.g., phosphorylation of MPK3/6, activation of the SA and JA pathways, as shown for direct activation of defense responses by ascr#18 in Manosalva et al. 2015; reference 34) in this sentence was premature. We have changed the sentence to: "Based on the finding that ascr#18 treatment increased accumulation of furanocoumarin in parsley, we hypothesized that ascr#18 may prime defense responses also in other plant species."

- Which ascr#18 concentration was used in the Arabidopsis root drench experiments? Why was this concentration chosen?

Response: A concentration of 1 μM was selected, based on our prior research demonstrating that it offers the maximum defense response and protection against pathogens in Arabidopsis.

- 1 μM Ascr#18 treated Arabidopsis seeds were inoculated at 5 weeks old. Why this concentration? And moreover, why this time-point? To add novelty to the paper, a time-course experiment would have be instrumental.

Response: In earlier work we showed that 1 μM of ascr#18 effectively enhances disease resistance of Arabidopsis (Manosalva et al., 2015; reference 34). Although we do not know whether measurements at other time points would result in even stronger effects, it should be noted that 4-5 weeks is the standard age used for pathogenicity assays in Arabidopsis by the research community.

- The pathogens, crops and ascr#18 concentrations used in this manuscript seem to be randomly chosen. It is not well-justified why they were selected.

Response: As explained above, we have extensive experience with selecting ascr#18 concentrations for different crops based on prior work. The pathogens were selected based on their economic significance, the high incidence of occurrence in the region where the trials took place, and their broader impact on agriculture.

- On page 7, the authors seem to suggest that histone acetylation is monitored by the FAIRE approach. However, in reality, euchromatin regions are picked-up by this technique. Euchromatin is modified by many more epigenetic modifications than just histone acetylation. This should be better explained.

Response: Thanks for this suggestion. We have revised the text which now reads "Priming-associated chromatin modifications are manifold and include changes in the methylation status of DNA and alterations in the methylation and acetylation status of specific lysine residues at the amino termini of histones H3 and H4, which reduces the interaction of nucleosome neighbors and lessens ionic DNA-histone interaction, thereby providing docking sites for regulatory proteins."

- In the field experiments, ascr#18 was sprayed on the plants. But all other experiments in this manuscript use seed or root treatment. Why foliar treatment in the field? Was the compound formulated to allow for example uptake in the leaves or rain-fastness? It is again remarkable that the treatments (number and dose) are varying between corn and soybean. Why?

Response: We tested several application methods in the lab, including root drenching, seed treatment, and foliar treatment, all of which proved effective. The foliar application is the most commercially relevant mode and therefore, we conducted field tests using it with a surfactant to enhance uptake and rain-fastness. We added this information to the Methods. As noted above the optimum ascr#18 concentration differs among plants, which is highest for soybean and

lower for corn. These differences in optimi were also reported in Klessig et al. (2019) *Journal of Phytopathology*, reference 32.

- Page 9: the authors state that there is 'sustained enhancement of immune gene expression'. This cannot be concluded based on only one time point.

Response: We agree with the reviewer. We have revised the sentence to "Pretreatment of *Arabidopsis* roots or seeds with ascr#18 leads to enhanced expression of a subset of immune gene upon pathogen challenge."

- Page 10: the paragraph starting with 'our results suggest that ...' is very confusing and not well-justified by the current dataset.

Response: This section explores the idea that conserved small molecule signatures, e.g., ascarosides as a hallmark of the presence of nematodes, HSLs as an indicator of the presence of a large group of bacteria, are broadly recognized by plants (and perhaps also animals). In conjunction with the previous finding that HSLs prime cell wall-associated defense in Arabidopsis that enhance immunity to pathogenic bacteria, our finding that ascarosides prime defense responses in many different plant species suggests the possibility that exposure to conserved small molecule signatures from a wide range of micro- and macrobiotic sources may play a significant role in shaping the immune systems of plants. We revised this paragraph for clarity.

- It has already been shown that non-plant-parasitic nematodes, such as entomopathogenic nematodes can also activate plant immune response (Shokofeeh Kamali et al, Molecular Ecology 2022). In light of this knowledge, I don't understand the statement that 'lack of defence priming by free-living nematodes could lead to increased plant susceptibility'. This statement is not supported by data provided in this manuscript, or in literature.

Response: Our data show that ascarosides prime plant defense responses. Ascarosides are produced in large amounts by free-living nematodes (also by entomopathogenic nematodes, see e.g. here https://www.nature.com/articles/s41598-020-62817-y) and thus under natural conditions plants are exposed to ascarosides in the soil. Therefore, it is a potential concern that lack of plant defense priming by free-living nematodes could result in increased disease susceptibility

- How many independent data points were evaluated in the infection experiments?

Response: Replicate numbers were added where missing (Figure 2, soybean) and are indicated in the Figure legends.

- How much RNA was used for the qRT-PCRs, in µg?- Using only one reference gene is a major problem in qRT-PCR. The MIQE guidelines (Bustin et al., 2009), which are nowadays

well-accepted and followed by numerous research groups, prescribe to use at least 2 reference genes for accurate quantification.

Response: 1 µg of RNA was used for the qRT-PCRs. As is most common in the plant sciences, the RT-qPCR data for all measured genes and treatments shown in the figures were normalized to *ACTIN2* mRNA transcript levels. Previous studies in the Conrath lab produced similar results regardless of whether normalization was done to *ACTIN2* or *UBIQUITIN5*. For examples, see Suppl Fig 1 in Jaskiewicz et al., 2011, EMBO rep, 12: 50-55 (http://dx.doi.org/10.1038/embor.2010.186) and Baum et al., 2020, Nat. Protocols 15:713-733 (https://doi.org/10.1038/nprot.2011.444)

Reviewer #3 (Remarks to the Author):

This well-written manuscript reports on an impressive amount of work from independent research groups. The paper describes the resistance-inducing activity of the nematode pheromone ascr#18, which was previously found to act as a nematode-associated molecular pattern (NAMP) with PTI-eliciting activity in Arabidopsis and to be perceived by the receptor-like kinase NILR1. The scientific novelty of the current study lies in the demonstration that treatment of taxonomically unrelated plants with relatively low concentrations of this compound induces long-lasting resistance against (hemi)biotrophic pathogens, which in some cases was maintained through the life cycle of plants and was associated with transcriptional priming of salicylic acid (SA)-inducible genes. The work combines high-quality experiments on cell suspension cultures and the genetic model plant Arabidopsis thaliana with field experiments of high translational value. The narrative of the Introduction and Results section is excellent and is supported by a detailed and clearly explained Methods section (barring some details – see my comments below). The Discussion is of equally high value but would perhaps have benefited from articulating the value of the ascr#18-Arabidopsis system as a new model system to study epigenetic plant immune memory.

In summary, I enjoyed reading this inspiring manuscript. Although it would be nice if the authors had experimental data to address some of my questions below, I must stress that these comments should not warrant major revisions. My questions come from a place of curiosity and admiration; in fact, as an editor of a plant journal, I would like to congratulate the editor on having the opportunity to publish a study of such outstanding quality!

Comments:

1. In Arabidopsis, seed treatment with ascr#18 induced long-lasting resistance in fully expanded leaves of 5-week-old plants, which was associated with chromatin relaxation in upstream promoter regions of SA-inducible genes (e.g., PR1, FRL1, WRKYs). I therefore wondered whether the authors have tested mutants in SA induction or perception (e.g., sid2, npr1) against Pseudomonas syringae pv. tomato DC3000 or downy mildew (Hyaloperonospora arabidopsidis, Hpa). This would establish a causal contribution of the transcriptional priming to the induced resistance. Additionally, as reported for other priming agents (e.g. BABA), it is possible that the contribution of SA to the induced resistance depends on the challenging pathogen.

Response: Thank you for this interesting question. We have conducted initial research with sid2-1 and npr1-1 mutants and found that the ascr#18 response was partially compromised

against Pst. However, the experiment was confounded because these mutant Arabidopsis plants are significantly more sensitive to Pst infection. We need to carry out additional experiments to determine the impact of these mutants on the ability of ascr#18 to induce or prime defense responses, but this is beyond the scope of the current study.

2. The long-lasting nature of ascr#18-induced resistance can best be explained by somatic transmission of an epigenetic priming signal. Indeed, the FAIRE-qPCR results support an epigenetic basis for the long-lasting induced resistance. In this context, I expected a short paragraph in the Discussion that articulates the value of the ascr#18-Arabidopsis system as a model for studying epigenetic immune memory. Additionally, it would be great if the authors included a bit more discussion about the nature of the epigenetic signal driving the somatic transmission of the priming. A brief glance at the integrated genome viewer (JBrowse; TAIR) shows that the five defence genes exhibiting long-term transcriptional priming do not have significant DNA methylation in their promoter regions. If reduced DNA methylation is the epigenetic signal responsible for somatic transmission of the priming (as proposed in previous studies), it would unlikely act via cis-acting mechanisms. Furthermore, I noticed that most of their gene bodies exhibit high levels of H2A.Z, H3K4me, and/or H3K27me3. Could these chromatin modifications play a role?

Response: We appreciate the reviewer's insightful comments regarding the potential epigenetic basis of ascr#18-induced resistance and the value of the ascr#18-Arabidopsis system as a model for studying epigenetic immune memory. In response, we have now added a paragraph to the Discussion (lines 311-324) that highlights the relevance of our system for investigating the molecular mechanisms underlying long-term immune priming. We thank the reviewer for this valuable suggestion and believe that these additions strengthen the manuscript by providing a broader perspective on the epigenetic regulation of defense priming.

3. The legend of Figure 3 for the FAIRE-qPCR results mentions "adjusted" p-values. According to the Methods section, these values are either based on ANOVA or two-tailed t-tests. However, it is unclear how the "adjusted" p-values were calculated, as only two conditions (control versus primed) are being compared. Assuming t-tests were performed, how did you account for the different promoter locations? Did you pool values from all promoter locations per treatment? While I have no doubts about the validity of the results, my impression is that simplistic linear models may not be the most appropriate approach to testing for statistical significance in this case. I suspect that more complex nested (generalized) linear models would be more appropriate.

Response: The reviewer is correct – "adjusted" does not make sense here. This was a remnant from an earlier draft of the manuscript where we had used a different statistical approach. As explained in the Methods, the *p*-values in this Figure were calculated using the modified Chi-squared method introduced by Hristova and Wimley (reference 91 in the revision, reference 84 in the original submission).

4. In the Methods section, please indicate the source of the ascr#18 compound.

Response: Thanks for pointing out this omission. We synthesized the ascr#18 in-house – this information has been added to the Methods section.

Response to reviewers' comments to COMMSBIO-24-8208A:
"**A conserved nematode pheromone primes plant immunity**"

We thank the three reviewers for their comments and helpful suggestions.

From the Editor's decision letter comments:

(…)

You will see from their comments below that while they find your work of considerable interest, some important points are raised. We are interested in the possibility of publishing your study in Communications Biology, but would like to consider your response to these concerns in the form of a revised manuscript before we make a final decision on publication.

We therefore invite you to revise and resubmit your manuscript, taking into account all of the referees' comments. In particular, please pay close attention to the concerns regarding (i) the proposed NILR1-dependent mechanism of priming, and (ii) the choice and validation of reference genes for RT-qPCR analysis. (…)

Response: To address these concerns, we have added new data further supporting the role of NILR1 for priming as well as data supporting the choice of reference genes for RT-qPCR analysis. Please see our detailed response to comments by new reviewer#2 below.

Reviewers' comments:

Reviewer #1 (Remarks to the Author):

The authors have addressed my questions, and I am satisfied. I now recommend the manuscript for publication.

Reviewer #3 (Remarks to the Author):

The authors have done a good job addressing my questions and I have no further questions/comments. I therefore strongly support publication of this manuscript without further delays.

Response: We thank these two reviewers for their useful suggestions.

Reviewer #4 (Remarks to the Author):

Reviewer #2 (Remarks to the Author): Review of COMMSBIO-24-8208-T

As the newly assigned reviewer, I have re-assessed the manuscript and generally concur with the observations of the previous reviewer. While the authors have made efforts to address the raised questions, the manuscript remains unclear in several areas. In certain instances, the authors appear to contradict their own prior observations, which may contribute to persistent confusion and ambiguity. My assessment is as follows:

- Many of the experiments presented are not novel, as they closely replicate findings from the authors' previous work. Additionally, the observed potency of ascr#18 in inducing defense genes appears inconsistent. For example, the results shown in Figure 1d differ from earlier publications: in the present study, ascr#18 alone does not significantly induce FRK1 and PR1, whereas previous work identified this as a key finding. The inconsistencies regarding defense

gene expression with ascr#18 alone and in combination with Pst infection, along with appropriate controls, warrant a detailed RNA-seq analysis.

Response:

We thank this reviewer for their detailed comments on our work.

"*Many of the experiments presented are not novel, as they closely replicate findings from the authors' previous work.*"

The current study demonstrates that ascr#18 strongly primes plant defense responses. Previous work showed increased induction of defenses in ascr#18-treated plants, but it remained unclear as to whether this is due to priming. Thus the current work provides a unique example for priming by a small molecule PAMP, at low nanomolar levels. To further strengthen evidence for priming, we added new data demonstrating that ascr#18-treatment of Arabidopsis systemically primes ROS responses to the bacterial elicitor flg22, and that ascr#18-mediated enhancement of flg22-induced ROS responses are NILR1-dependent. We further show that ascr#18 similarly primes flg22-induced ROS responses in tomato.

These new results show ascr#18-mediated priming is not restricted to transcriptional responses but also sensitizes early pattern-triggered immune signaling, as reflected by the enhanced flg22-induced ROS burst. This supports the idea that perception of nematode-derived ascarosides prepares plants for faster activation of multiple defense layers upon subsequent pathogen challenge.

"*Additionally, the observed potency of ascr#18 in inducing defense genes appears inconsistent.*"

Ascr#18, just like other priming agents such as salicylic acid, only partially induces plant defenses with a subset of genes being activated/induced directly to detectable levels, whereas other immune genes are only activated upon subsequent challenge by a pathogen (Sistenich et al., 2024). We directly address this point in the Discussion: "Pretreatment of *Arabidopsis* roots or seeds with ascr#18 enhances the expression of a subset of immune genes upon pathogen challenge. Notably, some immune genes exhibit modest direct induction by ascr#18, followed by a further increase in expression after pathogen challenge. Growth conditions can affect whether these genes exhibit only priming or both direct induction and priming. *WRKY6* and *WRKY29* illustrate this well; in the experiment shown in Figure 1, both genes are only primed, whereas in Figure 3 both are directly induced and primed."

Lastly, we agree that a detailed RNAseq analysis may reveal additional components of ascr#18-mediated signaling and priming pathways. In order to obtain meaningful results, this would require sampling at several different time points, ideally in different plants (e.g., Arabidopsis and tomato), and indeed our planned future work will investigate transcriptome-wide priming effects. The current manuscript provides the first direct evidence that a small molecule PAMP, ascr#18, provides long-lasting priming at low nanomolar concentrations, which results in strongly enhanced disease resistance in multiple crops.

- The experiments addressing the priming effect and the associated epigenetic mechanisms do not fully resolve the issue of novelty. The introduction of the concept of promoter opening in chromatin regions appears somewhat abrupt. The authors are encouraged to clarify the rationale for proposing that this epigenetic mechanism may be involved. Are there NGS datasets directly supporting this phenomenon? If not, it would be beneficial to explain in the introduction how chromatin remodeling could be relevant in this context. In lines 64–68, the manuscript describes how defense priming in general may be linked to epigenetic programming.

Are there literature sources that demonstrate or propose a critical role for epigenetic remodeling in ascr#18-mediated pathogen defense priming?

Response: We thank the reviewer for this helpful comment. Our intention was not to suggest that epigenetic regulation of defense priming *per se* is novel, as chromatin modifications have been implicated in several priming systems, as we mention in the Introduction. Rather, the novelty of our study lies in demonstrating that the nematode-derived signaling molecule ascr#18 induces defense priming that is associated with increased chromatin accessibility at specific defense gene promoters. Neither priming by ascr#18 nor the involvement of epigenetic mechanisms have been reported previously. We have revised the corresponding paragraph in the Introduction (lines 79-85) to make this clearer. Further, the paragraph on chromatin opening in the Results section includes brief mention of previous work demonstrating the relevance of chromatic opening for priming of defense responses.

-In line 69, the authors state that priming induces PPR accumulation. If this priming mechanism is proposed to be NILR1-dependent, it would be important to provide evidence of significant induction of NILR1 upon ascr#18 treatment, either through RT-qPCR or RNA-Seq (as suggested earlier), particularly given the absence of prior reports on this aspect. Furthermore, as NILR1 has been reported to be promiscuous in recognizing multiple pathogens, the authors should clarify how the requirement of the LRR-RLK NILR1 for ascr#18-mediated priming can be distinguished from shared downstream immune responses that occur across different pathogens following recognition. This point is especially relevant because Figure 3 shows reduced expression of defense genes (PR1, FRK1, and WKRY6) in Pst-treated plants in nilr1 mutants as well.

Response: We thank the reviewer for this thoughtful comment. We did not intend to suggest that ascr#18-mediated priming necessarily involves or requires transcriptional induction of NILR1 itself. Although priming in some systems has been associated with increased PRR abundance, enhanced receptor sensitivity or downstream signaling competence without detectable changes in receptor transcript levels is also well documented (see e.g., Gong, B.-Q. *et al.*, *Cell Host & Microbe* **26**, 810-822). Importantly, NILR1 has been shown to directly interact with ascr#18 and to be required for activation of downstream defense signaling (Huang et al. 2023), whereas PTI responses to other MAMPs, e.g., flg22, are not NILR1-dependent (Mendy et al., 2017). Our data (new Fig. 3b-d) is consistent with these earlier results, demonstrating that flg22 induces ROS to similar extents in Col-0, *nilr1-1*, and *nilr1-2* plants, but that ascr#18 treatment enhances the flg22-induced ROS response only in Col-0, but not in *nilr1-1*, and *nilr1-2* plants. Nonetheless, we agree that NILR1 may also contribute more broadly to immune responses against multiple pathogens. We have therefore clarified in the Discussion (lines 428-431) that our data support a requirement for NILR1 in ascr#18-mediated priming but do not imply strict stimulus specificity. Rather, NILR1 likely functions upstream in immune signaling pathways that are shared with other defense responses.

-The use of a single reference gene (ACTIN2) remains questionable. Citing similar results from other studies using different reference genes is not a sufficient justification. Please repeat the RT-qPCR with at least one additional reference gene and normalize data using the geometric mean of ACTIN2 and the additional gene(s), or provide evidence that ACTIN2 was the only stable reference under all conditions. In Manohar et al., 2020 (Nature Communications), UBQ10 was used. Please explain the rationale for switching to ACTIN2 and include a supplementary table with raw Ct values for ACTIN2 from this study and UBQ10 from the previous one to avoid confusion.

Response: We thank the reviewer for raising this important point. In the present study, gene expression data were normalized using ACTIN2, and, as requested, we have added a supplementary table (see new tabs in the “Supplementary Data 1” file) with raw Ct values for ACTIN2 from this study and UBQ10 from previous studies of the effects of ascr#18 on plants. To further address this reviewer’s concern regarding reference gene stability, we compared Ct values of ACTIN2 with two additional commonly used reference genes (UBIQUITIN5 and TUBULIN4) across different conditions in an earlier, unrelated Arabidopsis study that uses a similar experimental design. These analyses showed similar Ct behavior for all three genes, supporting the suitability of ACTIN2 as a stable reference gene under our experimental conditions. The corresponding Ct data are now provided as a reviewer-only Supplementary File S1.

Response to reviewers’ comments to COMMSBIO-24-8208B:
“**The conserved nematode pheromone ascr#18 primes plant immunity**”

We thank the reviewers for their evaluation and their positive comments.
